# Mortality among Care Home Residents in England during the first and second waves of the COVID-19 pandemic: an observational study of 4.3 million adults over the age of 65

**DOI:** 10.1016/j.lanepe.2021.100295

**Published:** 2022-01-10

**Authors:** Anna Schultze, Emily Nightingale, David Evans, William Hulme, Alicia Rosello, Chris Bates, Jonathan Cockburn, Brian MacKenna, Helen J Curtis, Caroline E Morton, Richard Croker, Seb Bacon, Helen I McDonald, Christopher T Rentsch, Krishnan Bhaskaran, Rohini Mathur, Laurie A Tomlinson, Elizabeth J Williamson, Harriet Forbes, John Tazare, Daniel Grint, Alex J Walker, Peter Inglesby, Nicholas J DeVito, Amir Mehrkar, George Hickman, Simon Davy, Tom Ward, Louis Fisher, Amelia CA Green, Kevin Wing, Angel YS Wong, Robert McManus, John Parry, Frank Hester, Sam Harper, Stephen JW Evans, Ian J Douglas, Liam Smeeth, Rosalind M Eggo, Ben Goldacre, David A Leon

**Affiliations:** a London School of Hygiene and Tropical Medicine, Keppel Street, London WC1E 7HT; b The DataLab, Nuffield Department of Primary Care Health Sciences, University of Oxford, OX26GG; c TPP, TPP House, 129 Low Lane, Horsforth, Leeds, LS18 5PX; d Department of Community Medicine, UiT The Arctic University of Norway, Tromsø, Norway; e International Laboratory For Population and Health, National Research University Higher School of Economics, Moscow, Russia

**Keywords:** Old Age Homes, Nursing Homes, COVID-19, Mortality, Electronic Health Records

## Abstract

**Background:**

Residents in care homes have been severely impacted by COVID-19. We describe trends in the mortality risk among residents of care homes compared to private homes.

**Methods:**

On behalf of NHS England we used OpenSAFELY-TPP to calculate monthly age-standardised risks of death due to all causes and COVID-19 among adults aged >=65 years between 1/2/2019 and 31/03/2021. Care home residents were identified using linkage to Care and Quality Commission data.

**Findings:**

We included 4,340,648 people aged 65 years or older on the 1st of February 2019, 2.2% of whom were classified as residing in a care or nursing home. Age-standardised mortality risks were approximately 10 times higher among care home residents compared to those in private housing in February 2019: comparative mortality figure (CMF) = 10.59 (95%CI = 9.51, 11.81) among women, and 10.87 (9.93, 11.90) among men. By April 2020 these relative differences had increased to more than 17 times with CMFs of 17.57 (16.43, 18.79) among women and 18.17 (17.22, 19.17) among men. CMFs did not increase during the second wave, despite a rise in the absolute age-standardised COVID-19 mortality risks.

**Interpretation:**

COVID-19 has had a disproportionate impact on the mortality of care home residents in England compared to older residents of private homes, but only in the first wave. This may be explained by a degree of acquired immunity, improved protective measures or changes in the underlying frailty of the populations. The care home population should be prioritised for measures aimed at controlling COVID-19.

**Funding:**

Medical Research Council MR/V015737/1

**Research in Context**

**Evidence before this study**

Residents of care homes in the UK and elsewhere are known to have been severely affected by the COVID-19 pandemic. In the UK this has been clearly demonstrated by very large increases in the number of excess deaths occurring in care homes in first and second waves 2020/21, and by studies in England, Scotland and Wales up to the summer of 2020. However, to date there have not been any large-scale studies of care home mortality in England over the first two pandemic waves that have been based on follow-up of care home residents regardless of whether they died where they lived or in hospital.

**Added value of this study**

Much of previously published literature on COVID-19 in care homes have focused on excess mortality, and data comparing mortality among care home residents to private home residents during the pandemic has not been published for England. Our study uses an address linkage to define a population of care home residents in England registered with GPs using the TPP EHR system and quantifies their mortality risk compared to individuals of a similar age residents in private homes between February 2019 and March 2021. We find that the first COVID-19 wave in the UK has had a disproportionate impact on care home residents. Age-standardised mortality risks were approximately 10-fold higher for care home residents compared to private home residents in the pre-pandemic period; this increased to approximately 18-fold during the peak of the first pandemic wave. However, during the second wave, mortality risks increased to the same proportional degree among care home residents and residents of private homes.

**Implication of all the available evidence**

Despite UK governmental policy aimed at protecting care homes, residents in England experienced disproportionately high mortality during the first COVID-19 pandemic wave. It is possible that some degree of immunity induced by infections in the first wave, improved protective measures or changes in the underlying frailty of the populations studied may have contributed to the absence of such an impact during the second wave. Our data supports targeting protective measures, including vaccinations, towards residents as well as ensuring social care staff have the resources required to implement infection control measures.

Alt-text: Unlabelled box

## Introduction

The COVID-19 pandemic has had a major adverse effect on the residents of care homes in the UK and in many other countries.^1^ By the end of the first wave in England and Wales (August 2020) the Office for National Statistics (ONS) estimated that almost a third of all deaths occurring among care home residents in the pandemic had been due to COVID-19.^2^ These 19 thousand COVID-19 deaths of care home residents accounted for approximately 40% of all COVID-19 deaths in England and Wales.^3^ However, this is likely to be an underestimate given the low levels of testing in care homes at the time. The Health Foundation estimated that there were approximately 10,000 additional so-called “excess” deaths among care home residents in England alone during the first wave.^3^ In addition, it has been found that the vast majority of excess care home deaths in England and Scotland occurred in care homes where there had been COVID-19 outbreaks.^4^^,^^5^

The impact of the COVID-19 pandemic on the risk of death among care home residents in England has not yet been comprehensively investigated and placed in the context of the mortality of people living in private residences, in part because of the absence of a national registry of care home residents. Working on behalf of NHS England, our aim was to provide the first direct estimates of mortality risks of care home residents compared to that of individuals in private residences across a period starting in February 2019 through waves 1 and 2 ending in March 2021. Adequate quantification of these differences is an essential component of learning the lessons of COVID-19.

## Methods

Our methods were developed to provide monthly updated estimates of the population at risk and deaths for residents in care homes and those in private households across our follow-up period from 1 February 2019 to 31 March 2021 in a large electronic health record database of patients registered with General Practices (GPs) in England.

### Data Source

Primary care records managed by the GP software provider TPP were linked to ONS death data through OpenSAFELY, a data analytics platform created by our team on behalf of NHS England to address urgent COVID-19 research questions (https://opensafely.org). OpenSAFELY provides a secure software interface allowing the analysis of pseudonymized primary care patient records from England in near real-time within the TPP Electronic Health Records (EHR) vendor's highly secure data centre, avoiding the need for large volumes of potentially disclosive pseudonymized patient data to be transferred off-site. This together with other technical and organisational controls, minimizes any risk of re-identification. Pseudonymized datasets from other data providers are securely provided to the TPP and linked to the primary care data. The dataset analysed within OpenSAFELY is based on 24 million people currently registered with GP surgeries using TPP SystmOne software. It includes pseudonymized data such as coded diagnoses, medications and physiological parameters. No free text data are included. Further details on our information governance can be found in the appendix, under information governance and ethics.

### Study Design and Population

We extracted 26 monthly cohorts of people aged 65 years or older with a valid address registered with a TPP practice on the 1^st^ of every month from 1^st^ February 2019 until 31^st^ March 2021. Valid address data is missing for a small proportion of individuals aged 65 years or older registered with TPP practices (1.1%).

### Study Measures

The exposure of interest was residency in a care or nursing home on the 1^st^ of each month. The identification of care homes in OpenSAFELY has been previously described.^6^ Briefly, the address an individual used to register with their GP was matched to the Care Quality Commission (CQC) registry of public and privately owned old-age care homes. Natural language processing was applied to the addresses to account for spelling inconsistencies, and data cleaning based on the number of residents at a given address was also undertaken. This process allowed us to assign to each individual their expected care home status at any point in time. Individuals who were not classified as being a care home resident were considered to be living in a private household, the latter referred to subsequently as private homes.

The outcome of interest was mortality captured by the Office for National Statistics (ONS). COVID-19 deaths were defined as having an underlying or secondary cause of death listed as COVID-19 (ICD-10 codes U07.1 or U07.2). Specific non-COVID-19 underlying causes of death of interest were also described: deaths due to cancer (ICD-10 chapter code C), cardiovascular disease (ICD-10 chapter I), respiratory disease (ICD-10 chapter J) and dementia (ICD-10 codes F00, F01, F02, F03 and G30). Deaths with any of these underlying causes but a secondary cause of death listed as COVID-19 were considered to be due to COVID-19.

The demographic and clinical characteristics considered were age, gender, self-reported ethnicity (5 categories), Nomenclature of Territorial Units for Statistics geographical region of the GP practice, quintile of index of multiple deprivation, stroke, dementia, diabetes, chronic kidney disease, cancer, chronic liver disease, chronic cardiac disease and chronic respiratory disease (https://github.com/opensafely/carehome-noncarehome-death-research/tree/master/codelists).

We defined the first pandemic wave as starting on 1^st^ February 2020 and lasting until 31^st^ August 2020, and the second wave as starting on 1^st^ September 2020 until the end of data availability as per previous studies.^7^

### Statistical Methods

Monthly mortality risks were calculated by totalling the number of deaths occurring during a given calendar month among people meeting the inclusion and exclusion criteria at the 1^st^ of that specific month (numerator) and dividing these by the number of individuals meeting the inclusion and exclusion criteria at the beginning of the interval (denominator). Relative risks were calculated by dividing the mortality proportions among care and/or nursing home residents by that among those living in private residences. To account for differences in the age of care home and private home residents, mortality risks for men and women were directly standardised to the European Standard (2013) population using five-year age-bands.^8^,^9^ Both crude risks and DSRs were scaled to a consistent month length of 30 days. Comparative Mortality Figures (CMF) were calculated by taking the DSR among care home residents and dividing these by the DSR among residents of private homes. Confidence intervals for the CMF were calculated using standard approaches.^10^

Data management was performed using OpenSAFELY tools in Python 3.8 and analyses carried out using R version 3.6.2. All of the code used for data management and analyses, as well as redacted mortality numerator and denominator data used for the plots, is available under open licenses for review and re-use at https://github.com/opensafely/carehome-noncarehome-death-research.

### Supplementary Analyses

In order to investigate whether the composition of the care home population changed over time we extracted information on the characteristics at the start of the first wave and then at the start of the second wave for all residents and new residents (defined as residents who were not resident in a care home a month prior to the extraction date) separately. We also plotted the prevalence of comorbidities in the population on a monthly basis. As the identification of care home residents based on address linkage is likely to miss some care home residents, we also repeated analyses additionally including those with who had ever had a code for care home residency in their medical record. This resulted in an increase of approximately 25,000 in the number of people classified as care home residents.^6^ We did not use this definition in our primary analyses, as GP coded events do not reflect the time-varying nature of care home residency and the accuracy of these codes is not known. We also estimated the monthly probability of individuals being tested for COVID-19 and the probability of being admitted to hospital using linked data from Second Generation Surveillance System (SGSS) dataset and the Secondary Uses Service (SUS) dataset by place of residence (care home or private home). Finally we undertook analyses of directly standardised risks of mortality stratified into two broad age groups (up to age 80, and 80+ years) and according to whether the care home of residence was or was not a nursing home.

### Role of the Funding Source

Funders had no role in the study design, collection, analysis, and interpretation of data; in the writing of the report; and in the decision to submit the article for publication.

## Results

### Population Characteristics

There were 4,340,648 individuals aged 65 years or older registered with TPP practices in the first monthly cohort extracted on 1st February 2019, and of these 95,215 (2.2%) were classified as resident in care or nursing homes. As shown in Table 1 residents in care homes were older than those in private homes (mean age 80 vs 75 years), more likely to be female (70% vs 54%) and much more likely to have been diagnosed with comorbidities, including dementia (59% vs 4%) and stroke (22% vs 6%). The size of the care home population did not change markedly over the period of observation (supplementary tables S1a-c; S4).

**Table 1:** Demographic and Clinical Characteristics of Care Home and Private Home Residents on the 1st of February 2019.

		Overall		Care or Nursing Home		Private Home	
		N	%	N	%	N	%
**Total**		4340648	100	95212	100	4245436	100
Care Home Type	Care Home	49227	1.13	49227	51.7		
	Care or Nursing Home^1^	2352	0.05	2352	2.47		
	Nursing Home	43633	1.01	43633	45.83		
	Private Home	4245436	97.81			4245436	100
Gender	Female	2338072	53.86	66574	69.92	2271498	53.5
	Male	2002576	46.14	28638	30.08	1973938	46.5
Age in Years	Mean, SD	75	8	86	8	75	8
Self - reported Ethnicity	Asian or British Asian	111186	2.56	556	0.58	110630	2.61
	Black	32879	0.76	437	0.46	32442	0.76
	Missing	1013993	23.36	25080	26.34	988913	23.29
	Mixed	11951	0.28	188	0.2	11763	0.28
	Other	26420	0.61	322	0.34	26098	0.61
	White	3144219	72.44	68629	72.08	3075590	72.44
Geographical Region	East	1023036	23.57	22328	23.45	1000708	23.57
	East Midlands	767248	17.68	17112	17.97	750136	17.67
	London	164809	3.8	1914	2.01	162895	3.84
	North East	204275	4.71	4087	4.29	200188	4.72
	North West	403745	9.3	9291	9.76	394454	9.29
	South East	320911	7.39	8152	8.56	312759	7.37
	South West	724881	16.7	15699	16.49	709182	16.7
	West Midlands	154436	3.56	3028	3.18	151408	3.57
	Yorkshire and The Humber	576444	13.28	13589	14.27	562855	13.26
	Missing	863	0.02	12	0.01	851	0.02
Quintile of Index of Multiple Deprivation (IMD)^2^	1 - Least Deprived	616788	14.21	17284	18.15	599504	14.12
	2	761404	17.54	19803	20.8	741601	17.47
	3	979568	22.57	20773	21.82	958795	22.58
	4	995263	22.93	19147	20.11	976116	22.99
	5 - Most Deprived	968925	22.32	17753	18.65	951172	22.4
	Missing	18700	0.43	452	0.47	18248	0.43
History of Stroke		289444	6.67	20884	21.93	268560	6.33
Dementia		212289	4.89	56433	59.27	155856	3.67
Diabetes		925887	21.33	22331	23.45	903556	21.28
Chronic Kidney Disease		445118	10.25	19579	20.56	425539	10.02
Cancer		642846	14.81	15424	16.2	627422	14.78
Chronic Liver Disease		36390	0.84	919	0.97	35471	0.84
Chronic Cardiac Disease		867578	19.99	28884	30.34	838694	19.76
Chronic Respiratory Disease		488040	11.24	12514	13.14	475526	11.2
New Resident^3^		4880	0.11	4880	5.13	0	0

1. “Care or Nursing Home” refers to residential homes for which the categorisation as a care or nursing home was uncertain.

2. Estimates of the IMD derived using the care home address

3. Not resident on the 1^st^ of the month prior

### Mortality Trends

**Figure 1:**
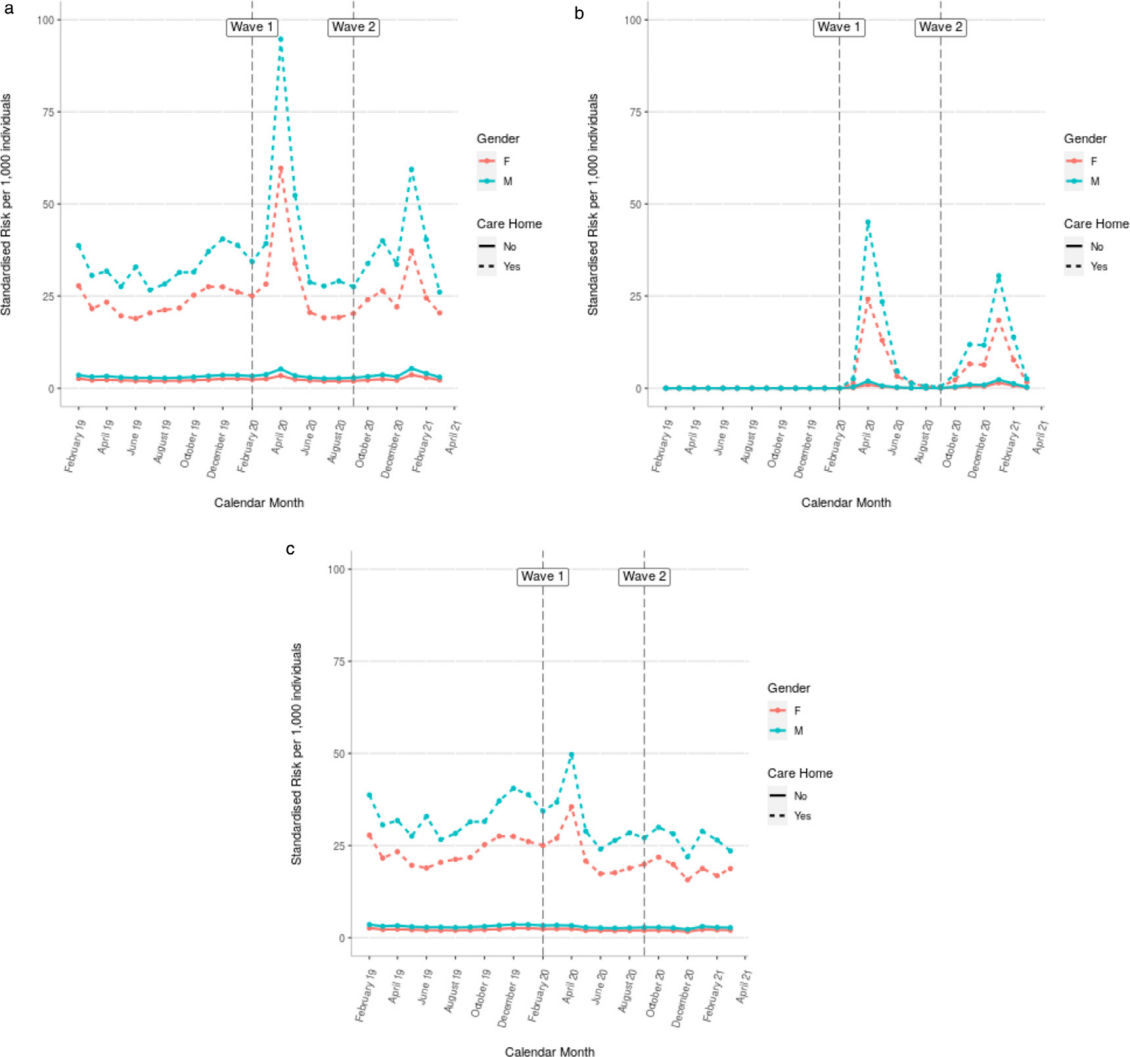
Age-standardised a) all-cause, b) COVID-19, and c) non-COVID-19 Monthly Mortality Risks over Time among Care and Private Home Residents by Gender.

Trends in age-standardised mortality risks across months among care home and private home residents according to gender are shown in Figure 1a–1c and supplementary materials table S2a-S2c. Among both men and women mortality risks increased appreciably during the first and second wave of the pandemic, peaking in April 2020. Relative to February 2019, at the peak of the first wave mortality risks in care homes increased by 115% among women and 147% among men. In contrast the increases over the same period were smaller for people in private homes: 30% for women and 47% for men (supplementary material table S2a). COVID-19 specific and non-COVID-19 mortality showed the same pattern of variation. It is striking that unlike in the first wave, there was no increase in non-COVID-19 risks in the second wave. As expected, the mortality risks among care home residents were appreciably higher compared to those in private homes both in the pandemic and pre-pandemic period.

**Figure 2:**
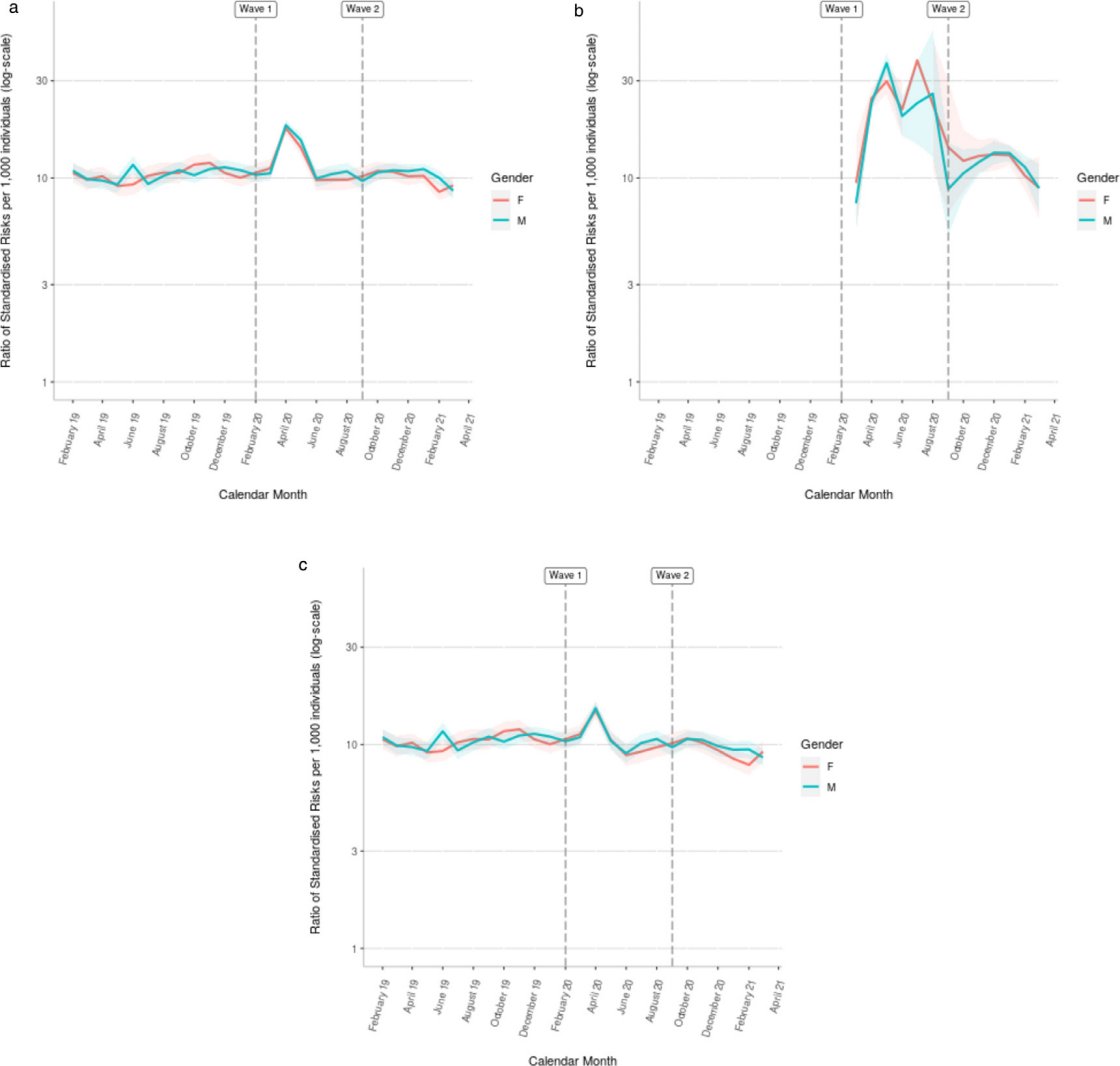
Comparative Mortality Figures (CMFs) Comparing Monthly a) all-cause, b) COVID-19, and c) non-COVID-19 Mortality over Time among Care Home versus Private Home Residents.

The comparative mortality figures, describing the relative increase in the mortality risk among care home residents compared to private home residents, are shown in Figure 2a – 2c and supplementary tables S3a-3c. Pre-pandemic care home residents had just over 10 times the risk of dying of any cause compared to private home residents (among women CMF = 10.59, (95%CI = 9.51, 11.81) and men CMF = 10.87 (9.93, 11.90)). By April 2020, this had increased to more than 17 times the mortality risk among residents of private homes of a similar age (women CMF = 17.57 (16.43, 18.79) men CMF = 18.17 (17.22,19.17)). By June 2020, the comparative mortality figures had returned to their pre-pandemic value. Of note, the comparative mortality figures did not increase during the second wave, despite a rise in the absolute age-standardised COVID-19 mortality risks (among women CMF at peak [October 2020] = 10.84 ((9.77, 12.03) and among men and CMF at peak [January 2021] = 11.08 (10.40, 11.79)).

### Age Differences in Mortality Trends

**Figure 3:**
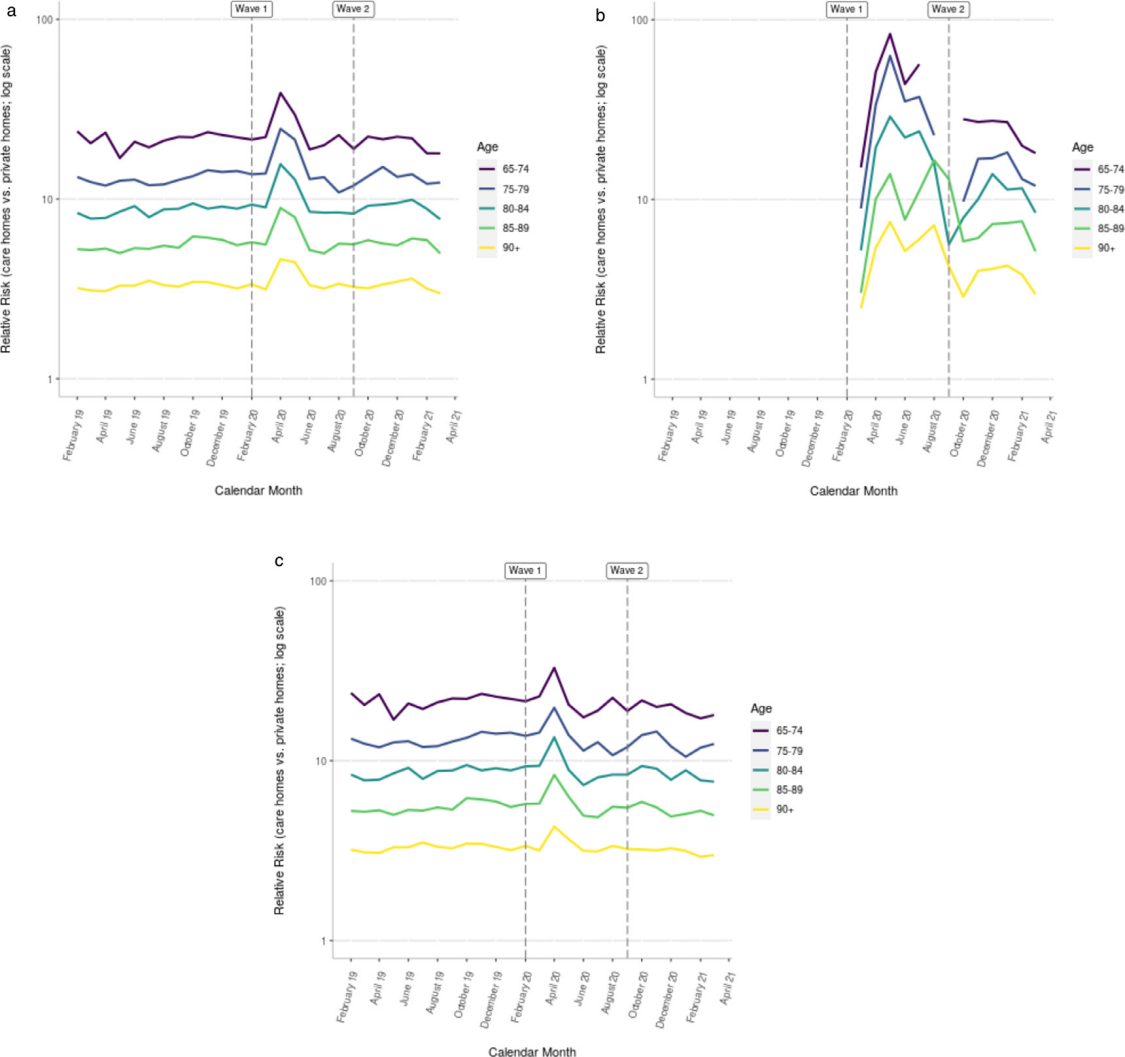
a-c. Relative Risk by age of (a) all-cause, (b) COVID-19*, and (c) non-COVID-19 Mortality over Time among Care Home versus Private Home Residents.

Relative mortality risks (care home vs private home) within narrow age-strata (Figure 3a-3c) showed a similar overall pattern to the overall age-standardised mortality risks. Mortality risks showed a striking tendency to decline with age, and the largest fluctuations over time were seen in the youngest care home residents. Trends were similar for all-cause, COVID-19 and non-COVID mortality.

### Proportional Trends in non-COVID causes of death

**Figure 4:**
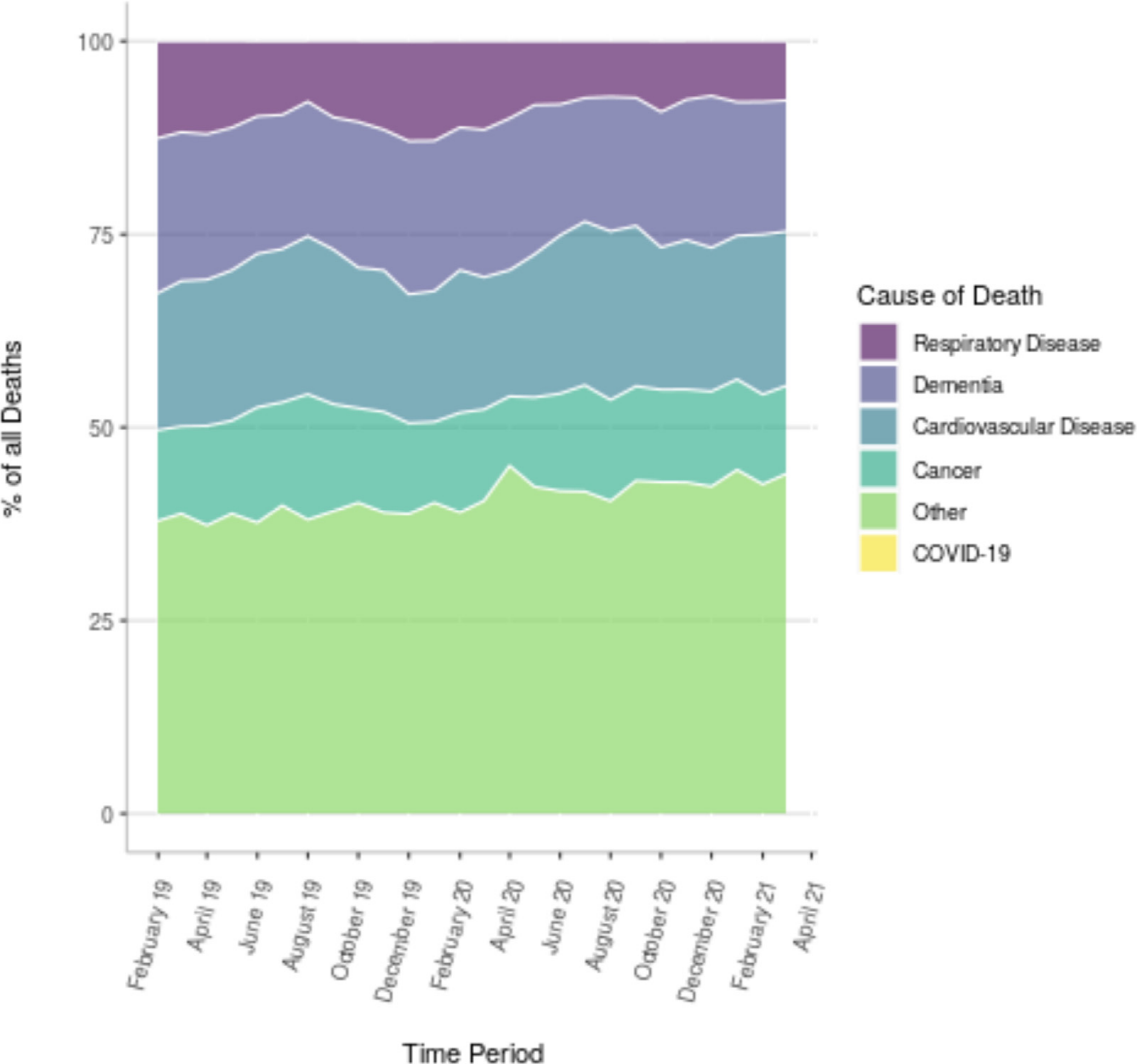
Percentage of non-COVID Deaths over Time among Residents of Care Homes.

The proportional cause-composition of non-COVID deaths among care home residents over time is shown in Figure 4. While this graph does not capture absolute changes in the rate of cause-specific deaths, it describes the composition of the non-COVID category of deaths over time. There were some slight changes in the cause-composition over the entire period of observation (e.g., an increase in the proportion of deaths due to ‘other’ causes and a slight decrease in the proportion of deaths due to respiratory disease), but these changes were gradual. Overall, there was no clear evidence of any particular cause becoming much more or less important during wave 1 despite the absolute increase in non-COVID risk at this time. A similar figure including COVID-19 deaths is provided in supplementary figure S13.

### Supplementary Analyses

Using a broader definition of care home residency did not change the overall trends observed (supplementary figures S7a-c S8a-c). Looking at the standardised risks of hospital admissions over time revealed a marked drop in the absolute age-standardised risk of all-cause and non-COVID hospitalisation for care home residents during the first wave (supplementary figures S10a-c). However, the relative probability of hospitalisation compared to private home residents did not decrease during either wave: it increased during the first wave and remained largely stable during the second wave (supplementary figures S11a-c). The probability of testing for COVID-19 increased for care home residents during both the first and second wave (supplementary figures S12a-b). Residents of care homes where nursing was provided experienced a higher standardised COVID-19 mortality risk than residents of non-nursing care home in the first but not the second wave (supplementary Figures 1a-c, 2a-c). In terms of age differences, trends described in Figure 3a-3c were similar when looking at age-standardised mortality risks stratified by age groups above and below 80 (supplementary figures 3a-3c, 4a-4c).

## Discussion

### Summary

Our main finding is that the relative mortality of people living in care homes compared to private homes increased during the first – but not the second – wave. This is novel and suggests that the mortality peak observed during the first wave may not have been inevitable. In the period before the pandemic, people aged 65 years or older living in care homes in England had approximately ten times higher mortality compared to those living in private homes. However, in the first wave of the pandemic this difference increased substantially to peak at an 18-fold difference, returning to the pre-pandemic 10-fold difference throughout the subsequent second wave. There was a substantial increase in non-COVID mortality among care home residents in the first wave only, although no evidence of any substantial shift in the distribution of non-COVID deaths by specific cause over the time-period studied.

### Comparison to Prior Literature

Hollinghurst et al, using data from Wales, took an approach similar to ours although their period of observation was shorter ending in mid-June 2020. They also found an increase in the relative risk of death during 2020 among care home residents compared to residents in private homes.^11^ However, the size of the relative difference in mortality risk between care and private homes they reported was smaller than ours as they adjusted for a range of comorbidities. Although we did not adjust for comorbidities in our analyses, there was no evidence of any substantive changes in comorbidity profiles over time (supplementary figure S9a-g, supplementary table S4) and adjustment would therefore be unlikely to affect our conclusions.

Outside of the UK, several studies have documented the impact of COVID-19 on care home residents. Fisman and colleagues quantified the risk of COVID-19 death among residents in long-term care (LTC) facilities in Canada. They found a significantly higher risk of COVID-19 death among residents, and the incidence rate ratio for death comparing LTC residents to private home residents increased dramatically during the pandemic – from 8.03 (95%CI:1.96 – 23.32) on 29 March 2020 to 87.28 (95%CI: 6.44 – 729.76) on 11 April 2020 albeit with wide confidence intervals for the latter time-point.^12^ Branden et al, studied the risk of COVID-19 death among adults aged 70 years or older during the first pandemic wave living in different residential contexts in Stockholm, Sweden. They found that adults in care homes had an increased risk of COVID-19 death (Hazard Ratio: 4.13, 95%CI: 3.49 - 4.90); this risk was substantially larger compared to that faced by adults living in private households irrespective of the age-structure of that household.^13^

Several studies have found that the significant all-cause excess mortality within care homes in the UK during the first wave of the pandemic was largely restricted to care homes with recorded COVID-19 outbreaks.^4^^,^^5^ This indicates that the excess mortality in care homes was primarily driven by COVID-19, irrespective of which cause of death was listed on the death certificate. Such under-recording of COVID-19 deaths could have occurred if there was an initial reluctance to list COVID-19 as a cause of death in the absence of a positive laboratory test result. Our data are consistent with this observation. We observed a marked increase in non-COVID-19 deaths only in the first, but not the second wave. An alternative explanation for the first wave excess deaths and increased non-COVID-19 mortality risk in care homes is that this could have been a consequence of lockdown with increased social isolation and loneliness (relatives were unable to visit) and reduced access to wider health service interventions. However, although the second-wave lockdown was associated with many of these same privations we did not see any increase in non-COVID mortality in care homes in the second wave.

Our finding that care home residents had an approximately 10-fold higher risk of death before the pandemic is supported by previous studies,^14^^,^^15^ reflecting the fact that poor physical and functional health is one key reason why people may end up living in a care home.

### Strengths and Limitations

Our study is the first large-scale analysis of mortality risks in England according to place of residence (care home vs private home) that includes a substantial pre-pandemic period and extends to cover both first and second waves up to the end of March 2021. The only national data pertaining to mortality in care homes is produced by the ONS but is limited by the fact that it is only based on place of death. It does not provide estimates of death rates because it lacks denominators, and over the course of the pandemic, there will have been changes in the probability of people being admitted to hospital or discharged to care homes. Our analysis, in contrast, compares absolute mortality rates according to residential status, regardless of place of death. Our study was one of the largest analyses of care home residents to date. TPP covers the medical records of approximately a third of the English population. 425,408 individuals are estimated to live in care homes in England:^16^ our study therefore includes approximately a quarter of all care home residents in England.

The main potential weakness of our analysis is that all of the methods available to identify care home residents in UK using electronic healthcare records are subject to limitations.^6^^,^^17^^,^^18^ We used an address linkage with CQC data. Although this is expected to have a high positive predictive value,^17^^,^^18^ the prevalence of care home residency is approximately a third lower than expected based on estimates from the 2011 census.^19^ To partially address this we conducted a post-hoc analysis which additionally used medical codes to identify care home residents, however, this did not impact the main trends observed (supplementary figures S7a-c, S8a-c), although the accuracy of using of medical codes for identifying care home residents is not known^6^ and some misclassification is likely. If the misclassification of care home residency depends on patient characteristics which influence a persons’ mortality risk, the direction of the resulting bias is hard to predict. However, we would expect the misclassification of residency status to affect our estimates of relative mortality risk in a largely similar manner over time, and thus the effect on our main conclusions concerning time trends may be relatively small. It should also be noted that whilst the care home linkage in OpenSAFELY-TPP can be used to identify whether an individual was likely to be resident in a care home at any given point, household level information is not available over time. Data on care home characteristics, such as ownership or CQC rating, was also not available.

TPP only covers a proportion of English residents, concentrated in the East of England, with lower representation of GP surgeries in London.^20^ Nevertheless, it is difficult to imagine how the scale of the mortality differences observed here could be restricted to TPP practices. Although there may be some differences in the trends observed across the UK, our data are consistent with that from Wales and Scotland.^4^^,^^11^ and the age and gender distribution of care home residents included in our study is similar to that described previously in studies based on different EHR software.^15^^,^^21^ Nevertheless, it is important to bear the representativeness of the TPP source population in mind when interpreting the results. Detailed comparisons between the characteristics of patients enrolled at TPP practices and the general population, as derived from census estimates, are currently ongoing.

### Interpretation and Policy Implications

In contrast to the first wave, the rise in mortality risks during the second pandemic wave affected private and care home residents to the same proportional degree and absolute mortality risks were lower compared to the first peak. Although our descriptive analysis cannot identify what has caused these mortality differences, potential explanations include differences in the measures taken to protect care home residents, changes in the underlying demographics of the population, or changes in the immunity of care home residents.

Several preventative measures were introduced in England between the first and the second wave. Initial measures focused on testing of staff and residents, provision of personal protective equipment (PPE),^22^ and visits were effectively banned with the announcement of the first national lockdown on 23 March 2020.^23^ However, the UK governments’ COVID-19 action plan for adult social care was not published until 15 April 2020.^22^, and there were widespread reports of limited availability of both testing and personal protective equipment (PPE) during the first wave.^24^ A lack of PPE has since been linked to higher case numbers once an outbreak has occurred.^25^ Several studies have also highlighted the importance of supporting staff to manage the risk of COVID-19 outbreaks, with numbers of staff not directly involved in the care of residents,^25^ not paying statutory sick pay, use of agency staff as well as lower staff-to-bed ratios^26^ all linked to a higher risk of COVID-19 outbreaks. A policy of regular testing of staff and residents, regardless of symptoms, was announced on the 3^rd^ of July 2020 - after the first wave.^22^ Willingness or ability to admit care home residents compared to private home residents may also have differed between the first and second wave, and this may in part explain mortality differences between different waves. Similar to other authors^27^ we found a marked decrease in the probability of all-cause and non-COVID admissions during the first wave among residents both of care homes and private homes, although it is noteworthy that the probability of total, COVID-19 and non-COVID hospital admission increased for care home residents compared to private home residents during the first wave. However, these analyses are hard to interpret as they do not capture the underlying clinical need for hospital admission or testing in the different populations. The increases in relative probability of admission for care home residents compared to private home residents could reflect the greater infection burden in care homes, and it is not possible to determine whether admissions met the healthcare needs of the population from this data.

In interpreting our results, particularly the differences between the first and second waves, it is necessary to consider that there may have been important differences in the care home populations over the pandemic. Most importantly, the very high mortality in care homes in the first wave may have induced a subsequent, transient short-term mortality displacement. We investigated this so-called ‘harvesting’^28^ effect . However, our supplementary analyses to detect any changes in the characteristics of care home residents over time failed to find any substantial support for this possibility. The age and gender composition, as well as the prevalence of key comorbidities appeared similar at the start of the first and second wave; with almost no change in the prevalence of dementia although a small increase in the prevalence of diabetes (from 24.8% to 25.4%). Looking at monthly trends in the comorbidity prevalence (supplementary figures S9a-g) did not reveal any significant changes in the comorbidity profile.

It is possible that the clinical data that we had available may not have been fully captured some of the more subtle changes in frailty profile that might have occurred due to mortality displacement following the first wave. We might expect residents who survived the first wave to represent a ‘survivor’ population, and those who died to have been replaced by younger, fitter residents with a lower mortality. Such demographic changes would be expected to at least partially reduce the mortality risk among care home residents during the second wave. In our data, non-COVID-19 deaths did not return to their pre-pandemic peak during the analysis period, further supporting the hypothesis that there has been some mortality displacement, despite this not being visible when looking at selected characteristics only. Changes in the resident population may also have caused changes in the occupancy rates within different care homes. Crowding in nursing homes has been linked to increased COVID-19 mortality risks,^29^ however, we were not able to describe how occupancy rates varied over time in our cohort due to a lack of data on household-level information over time.

Immunity among survivors acquired in the first wave may also have contributed to slowing the spread of the infection during the second wave. The VIVALDI study of 100 long-term care facilities in England found an antibody prevalence as high as 33% among residents in June 2020, with the presence of IgG antibodies strongly reducing the risk of reinfection among residents during the second wave.^30^ The UK vaccination program may also have contributed in part, although the mortality peak in January 2021 occurred before all care home residents in the UK had been vaccinated.^31^ Due to the time taken for immunity to develop, factors in addition to the vaccination programme are likely to have played a role. Finally, the variant strain of COVID-19 differed between the pandemic waves, with the more virulent variant of concern (VOC) SARS-CoV-2 B.1.1.7 dominant during the second wave. However, as age and comorbidity risk factors appear similar for non-VOC and VOC outcomes,^32^ we would expect this to affect care home and non-care home residents to the same degree and therefore not influence the relative mortality risk.

### Further Research

It is a priority to understand in more depth what might have caused the reduction in the relative mortality risk of care home residents during the second wave, and ongoing evaluations of different preventative measures should continue to be a priority area for future research. Such studies are possible by utilising observational data with information on care home characteristics, and comparing the risk of infection either at the individual or care home level according to whether particular measures were reported. This is an approach taken by a number of authors.^25^^,^^26^^,^^33^ Finally, although we believe that the identification of care home residents through an address linkage is a strength of the study, we also recognise that this will misclassify some individuals as residents in private homes. As others have argued,^34^^,^^35^ we strongly believe that the development of data infrastructure that can identify spells of care home residence should be a priority in order to allow the healthcare needs of this vulnerable population to be comprehensively characterised.

## Conclusion

Our analysis highlights the stark impact of the COVID-19 pandemic on care homes in England, with residents suffering a disproportionately increased mortality risk during the first wave compared to individuals of a similar age living in private residences. Although absolute mortality risks increased during the second wave, these remained below the first wave peak, and the relative mortality risk of care home residents compared to individuals living in private residences remained unchanged – possibly reflecting preventative measures such as increased testing and infection control measures within care homes, high levels of pre-existing immunity or changes in the demographics of the care home population.

## Contributions

Contributions are as follows: Conceptualization DL, LS, AS, RE Data curation CB JP JC SH SB DE PI CM RMM; Formal Analysis AS EN Funding acquisition BG LS; Information governance AM BG CB JP; Methodology AS, DL, EN, RE, CB, JC, RMM, WH Ethics approval HC EW LS BG; Project administration AS; Resources BG LS; Software SB DE PI AJW CM CB FH JC SH GH, SD, TW, LF, AG; Supervision DL LS BG Visualisation AS Writing (original draft) AS, DL Writing (review & editing) AS, EN, DE, WH, AR, CB, JC, BMK, HC, CEM, RC, SB, HIM, CTR, KB, RM, LAT, EJW, HF, JT, DG, AJW, PI, NJDV, AM, GH, SD, TW, LF, AG, KW, AYSW, RMM, JP, FH, SH, SJWE, IJD, LS, RE, BG, DL

## Data Availability

Access to the underlying identifiable and potentially re-identifiable pseudonymised electronic health record data is tightly governed by various legislative and regulatory frameworks, and restricted by best practice. The data in OpenSAFELY is drawn from General Practice data across England where TPP is the data processor. TPP developers initiate an automated process to create pseudonymised records in the core OpenSAFELY database, which are copies of key structured data tables in the identifiable records. These pseudonymised records are linked onto key external data resources that have also been pseudonymised via SHA-512 one-way hashing of NHS numbers using a shared salt. DataLab developers and PIs holding contracts with NHS England have access to the OpenSAFELY pseudonymised data tables as needed to develop the OpenSAFELY tools. These tools in turn enable researchers with OpenSAFELY data access agreements to write and execute code for data management and data analysis without direct access to the underlying raw pseudonymised patient data, and to review the outputs of this code. All code for the full data management pipeline—from raw data to completed results for this analysis—and for the OpenSAFELY platform as a whole is available for review at github.com/OpenSAFELY.

OpenSAFELY is currently piloting the onboarding of external users, and more information can be found here: https://www.opensafely.org/onboarding-new-users/.

## Funding

This work was supported by the Medical Research Council MR/V015737/1. TPP provided technical expertise and infrastructure within their data centre *pro bono* in the context of a national emergency.

BG's work on better use of data in healthcare more broadly is currently funded in part by: NIHR Oxford Biomedical Research Centre, NIHR Applied Research Collaboration Oxford and Thames Valley, the Mohn-Westlake Foundation, NHS England, and the Health Foundation; all DataLab staff are supported by BG's grants on this work. LS reports grants from Wellcome, MRC, NIHR, UKRI, British Council, GSK, British Heart Foundation, and Diabetes UK outside this work. AS and JT are employed by LSHTM on fellowships sponsored by GSK. KB holds a Sir Henry Dale fellowship jointly funded by Wellcome and the Royal Society (107731/Z/15/Z). HIM is funded by the National Institute for Health Research (NIHR) Health Protection Research Unit in Immunisation, a partnership between Public Health England and LSHTM. AYSW holds a fellowship from BHF. ID holds grants from NIHR and GSK. RM holds a Sir Henry Wellcome Fellowship funded by the Wellcome Trust (201375/Z/16/Z). HF holds a UKRI fellowship.

The views expressed are those of the authors and not necessarily those of the NIHR, NHS England, Public Health England or the Department of Health and Social Care.

## Information governance and ethical approval

NHS England is the data controller for OpenSAFELY-TPP; TPP is the data processor; all study authors using OpenSAFELY have the approval of NHS England. This implementation of OpenSAFELY is hosted within the TPP environment which is accredited to the ISO 27001 information security standard and is NHS IG Toolkit compliant;^36^, ^37^

Patient data has been pseudonymised for analysis and linkage using industry standard cryptographic hashing techniques; all pseudonymised datasets transmitted for linkage onto OpenSAFELY are encrypted; access to the platform is via a virtual private network (VPN) connection, restricted to a small group of researchers; the researchers hold contracts with NHS England and only access the platform to initiate database queries and statistical models; all database activity is logged; only aggregate statistical outputs leave the platform environment following best practice for anonymisation of results such as statistical disclosure control for low cell counts.^38^

The OpenSAFELY research platform adheres to the obligations of the UK General Data Protection Regulation (GDPR) and the Data Protection Act 2018. In March 2020, the Secretary of State for Health and Social Care used powers under the UK Health Service (Control of Patient Information) Regulations 2002 (COPI) to require organisations to process confidential patient information for the purposes of protecting public health, providing healthcare services to the public and monitoring and managing the COVID-19 outbreak and incidents of exposure; this sets aside the requirement for patient consent.^39^

Taken together, these provide the legal bases to link patient datasets on the OpenSAFELY platform. GP practices, from which the primary care data are obtained, are required to share relevant health information to support the public health response to the pandemic, and have been informed of the OpenSAFELY analytics platform.

This study was approved by the Health Research Authority (REC reference 20/LO/0651) and by the LSHTM Ethics Board (reference 21863).

## Guarantor

BG/LS is guarantor.

## Declaration of interests

BG has received research funding from the Laura and John Arnold Foundation, the NHS National Institute for Health Research (NIHR), the NIHR School of Primary Care Research, the NIHR Oxford Biomedical Research Centre, the Mohn-Westlake Foundation, NIHR Applied Research Collaboration Oxford and Thames Valley, the Wellcome Trust, the Good Thinking Foundation, Health Data Research UK (HDRUK), the Health Foundation, and the World Health Organisation; he also receives personal income from speaking and writing for lay audiences on the misuse of science. IJD has received unrestricted research grants and holds shares in GlaxoSmithKline (GSK). RM reports personal fees from AMGEN, outside the submitted work. All other authors have nothing to declare.

## Appendix Supplementary materials

Image, application 1

Image, application 2
