# Supplementary material for: Mortality among Care Home Residents in England during the first and second waves of the COVID-19 pandemic: an observational study of 4.3 million adults over the age of 65
Source: Lancet Reg Health Eur. 2022 Jan 10;14:100295. doi: 10.1016/j.lanepe.2021.100295 (PMC8743167; doi:10.1016/j.lanepe.2021.100295)
Supplement: Supplementary file 1 [file mmc1.docx]

**Supplementary Data: Mortality among Care Home Residents in England during the first and second waves of the COVID-19 pandemic: an observational study of 4.3 million adults over the age of 65**

## Note: key figures and tables referenced in text are provided formatted in this supplementary data, however, all raw output – redacted for numbers <=5 - is also available on github: <https://github.com/opensafely/carehome-noncarehome-death-research/tree/master/released_outputs/output/tables>

Table of Contents

[Supplementary Data 1](#_Toc82523866)

[Figure S1a-c. Age-standardised a) all-cause, b) covid and c) non-covid mortality risks according to care home type, among men. 2](#_Toc82523867)

[Figure S2a-c Age-standardised a) all-cause, b) covid and c) non-covid mortality risks according to care home type, among women. 3](#_Toc82523868)

[Figure S3a-c. Age-standardised a) all-cause, b) covid and c) non-covid mortality risks according to age group, among men 4](#_Toc82523869)

[Figure S4a-c. Age-standardised a) all-cause, b) covid and c) non-covid mortality risks according to age group, among women 5](#_Toc82523870)

[Figure S5a-c. Comparative mortality figures for a) all-cause, b) covid and c) non-covid mortality according to age group, among men 6](#_Toc82523871)

[Figure S6a-c. Comparative mortality figures for a) all-cause, b) covid and c) non-covid mortality according to age group, among men 7](#_Toc82523872)

[Figure S7a-c. Age-standardised a) all-cause, b) covid and c) non-covid mortality risks using an expanded definition of care home residency 8](#_Toc82523873)

[Figure S8a-c. Comparative mortality figures for a) all-cause, b) covid and c) non-covid mortality using an expanded definition of care home residency 9](#_Toc82523874)

[Figure S9a-f. Prevalence of different comorbidities over time 10](#_Toc82523875)

[Figure S10a-c. Age-standardised relative a) all-cause, b) covid and c) non-covid admission probabilities 12](#_Toc82523876)

[Figure S11a-c. Age-standardised relative a) all-cause, b) covid and c) non-covid admission probabilities 13](#_Toc82523877)

[Figure S12a-b. Age-standardised COVID-19 a) testing probability and b) relative testing probability 14](#_Toc82523878)

[Figure S13. Proportion of care home deaths over time, by cause including COVID-19 15](#_Toc82523879)

[15](#_Toc82523880)

[Table S1a. Crude all-cause mortality risks by age-group 16](#_Toc82523881)

[Table S1b. Crude COVID mortality risks by age-group 21](#_Toc82523882)

[Table S1c. Crude non-COVID mortality rates by age-group 26](#_Toc82523883)

[Table S2a. Age-standardised all-cause mortality risks by gender 31](#_Toc82523884)

[Table S2b. Age-standardised COVID mortality risks by gender 33](#_Toc82523885)

[Table S2c. Age-standardised non-covid mortality risks by gender 35](#_Toc82523886)

[Table S3a. Comparative Mortality Figure (all-cause) comparing Care Homes to Private Homes, by gender 37](#_Toc82523887)

[Table S3b. Comparative Mortality Figure (covid) comparing Care Homes to Private Homes, by gender 39](#_Toc82523888)

[Table S3c. Comparative Mortality Figure (non-covid) comparing Care Homes to Private Homes, by gender 41](#_Toc82523889)

[Table S4. Demographic and Clinical Characteristics of Care Home Residents over time 43](#_Toc82523890)

[Table S5. Demographic and Clinical Characteristics of New Care Home Residents at start of 1st and 2nd Wave 45](#_Toc82523891)

### **Figure S1a-c. Age-standardised a) all-cause, b) covid and c) non-covid mortality risks according to care home type, among men.**

1. b) c)

**
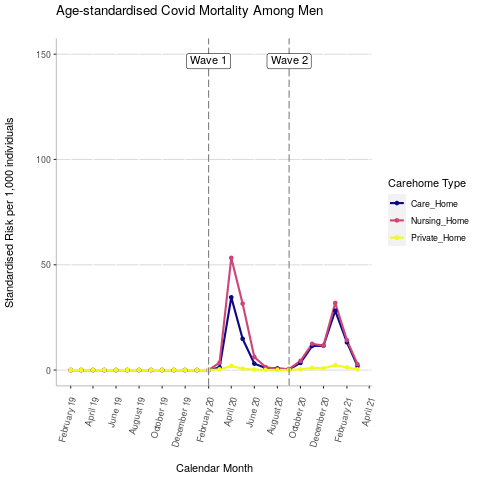

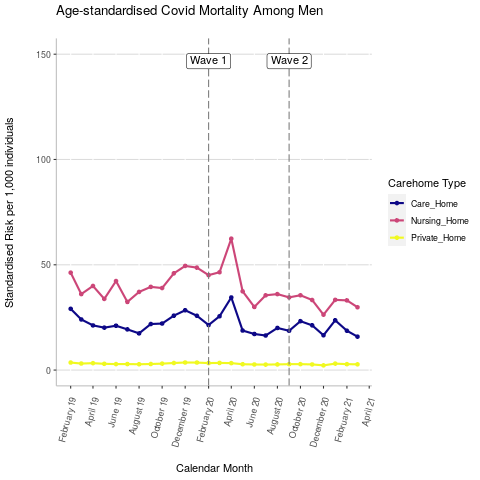

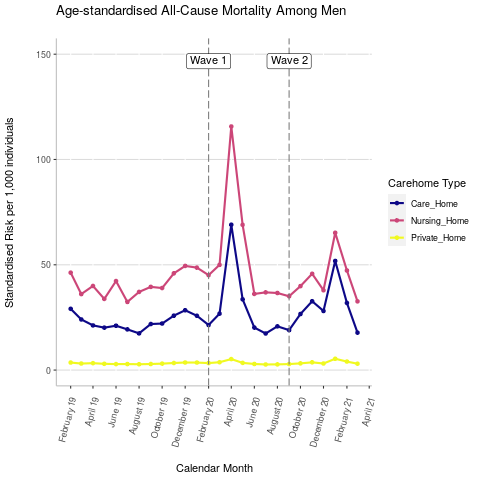
**

###

### **Figure S2a-c Age-standardised a) all-cause, b) covid and c) non-covid mortality risks according to care home type, among women.**

1. b) c)

**
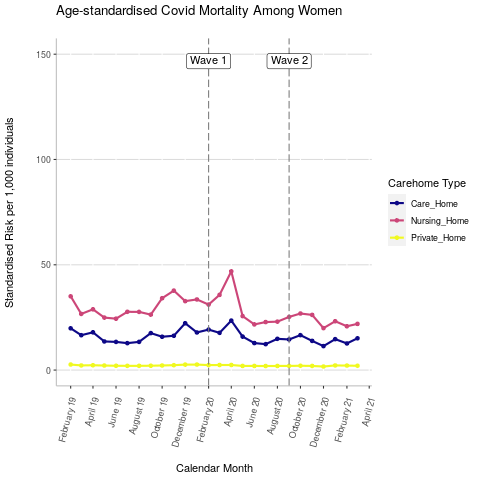

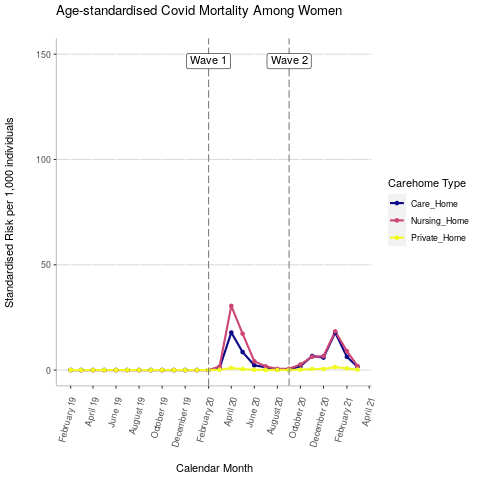

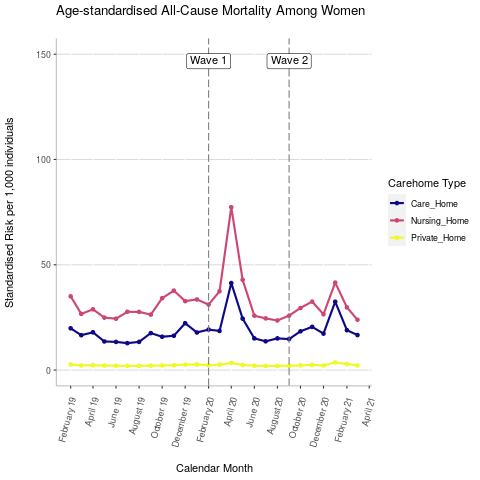
**

### **Figure S3a-c. Age-standardised a) all-cause, b) covid and c) non-covid mortality risks according to age group, among men**

1. b) c)

**
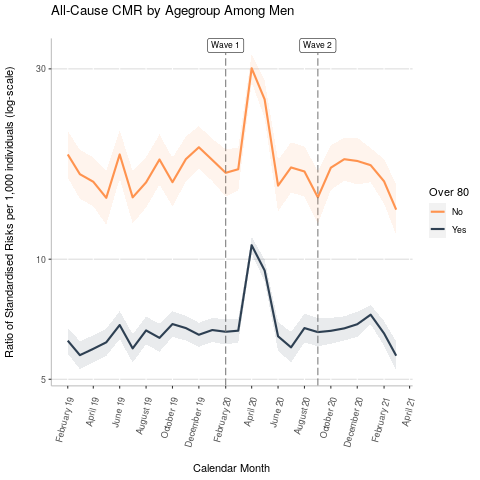

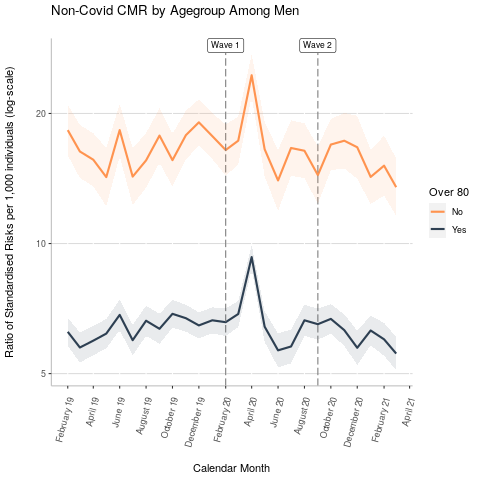

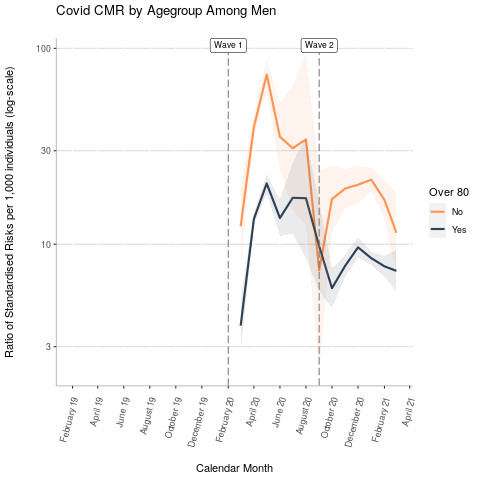
**

### **Figure S4a-c. Age-standardised a) all-cause, b) covid and c) non-covid mortality risks according to age group, among women**

1. b) c)


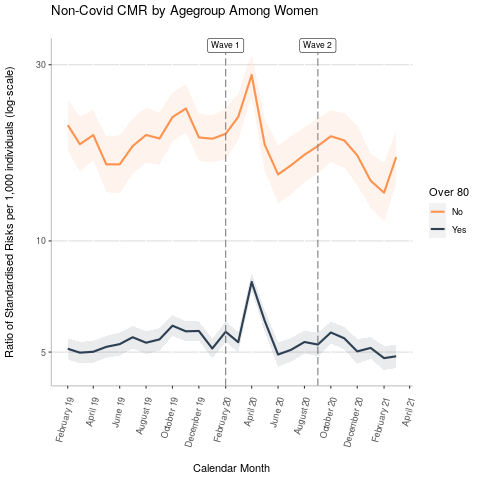

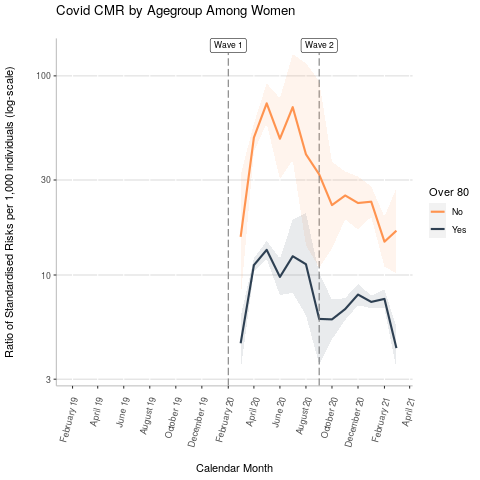

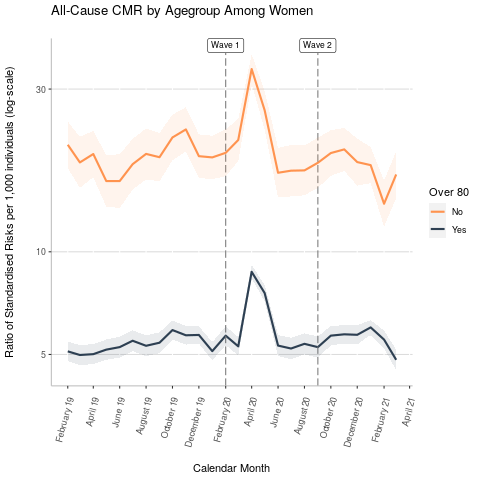


### **Figure S5a-c. Comparative mortality figures for a) all-cause, b) covid and c) non-covid mortality according to age group, among men**

1. b) c)


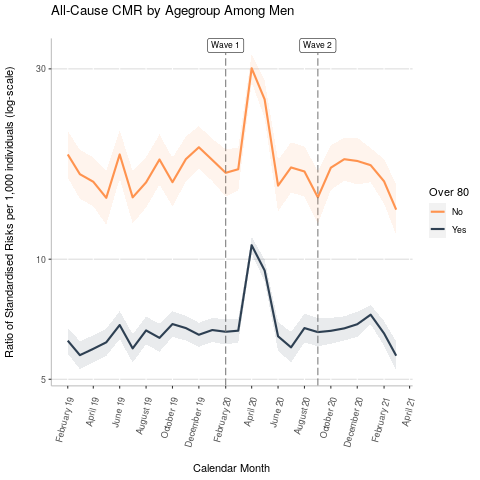

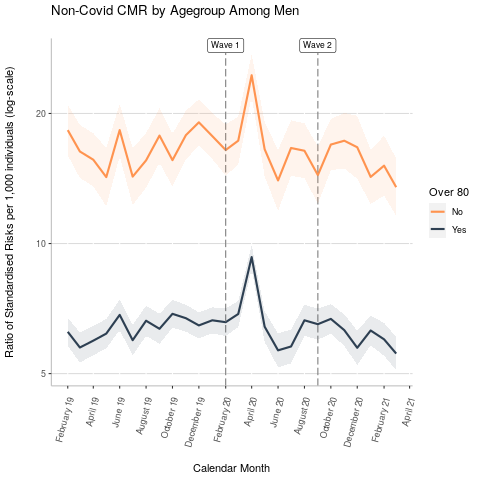

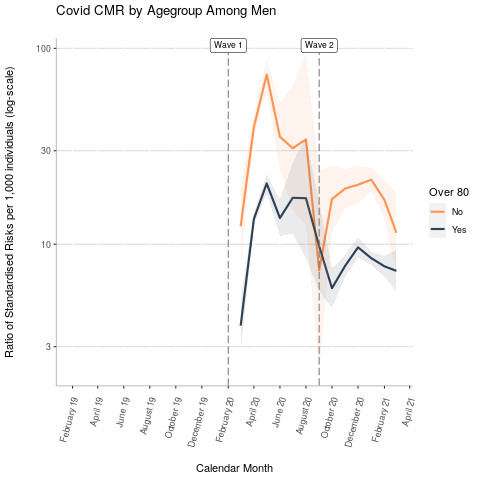


### **Figure S6a-c. Comparative mortality figures for a) all-cause, b) covid and c) non-covid mortality according to age group, among men**

1. b) c)


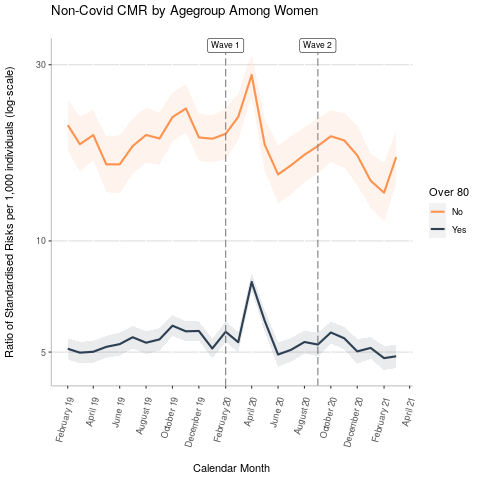

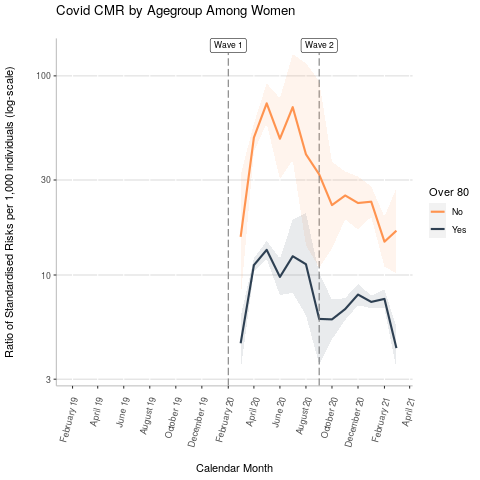

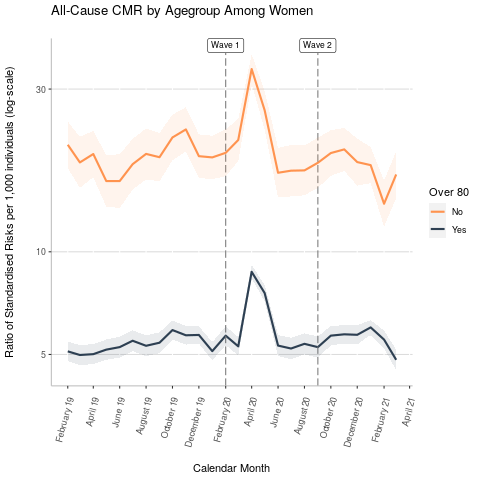


### **Figure S7a-c. Age-standardised a) all-cause, b) covid and c) non-covid mortality risks using an expanded definition of care home residency**

1. b) c)

**
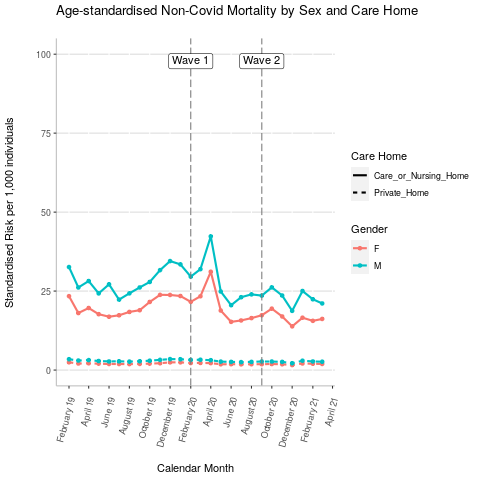

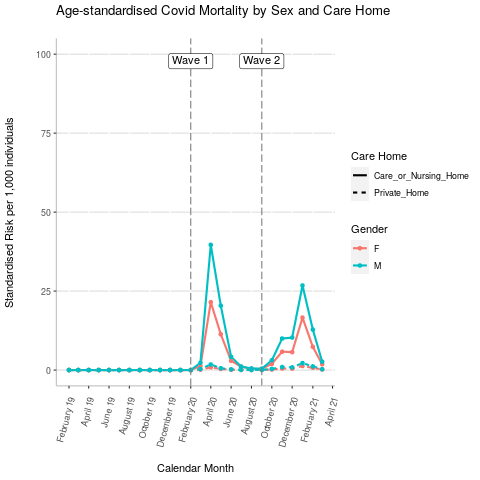

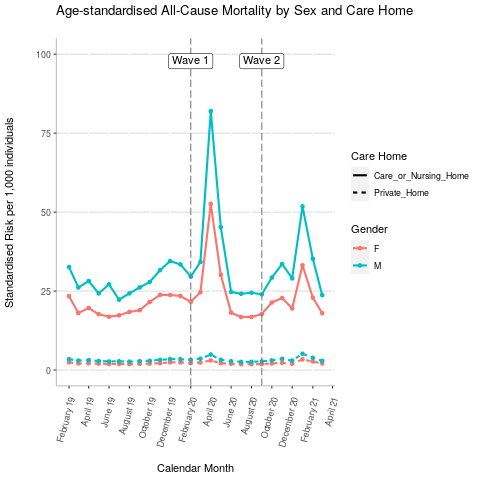
**

### **Figure S8a-c. Comparative mortality figures for a) all-cause, b) covid and c) non-covid mortality using an expanded definition of care home residency**

1.
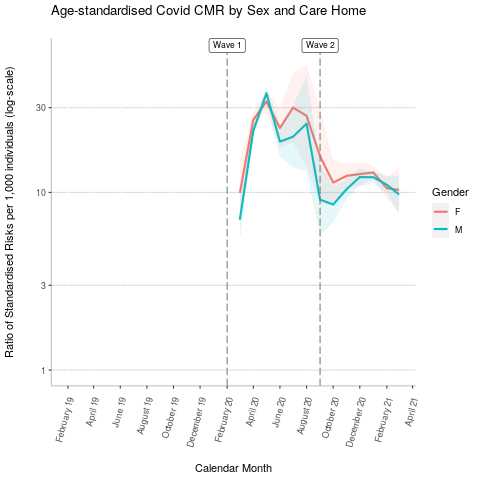
 b) c)


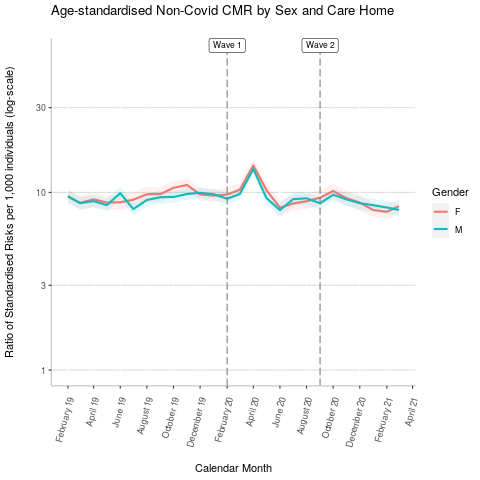

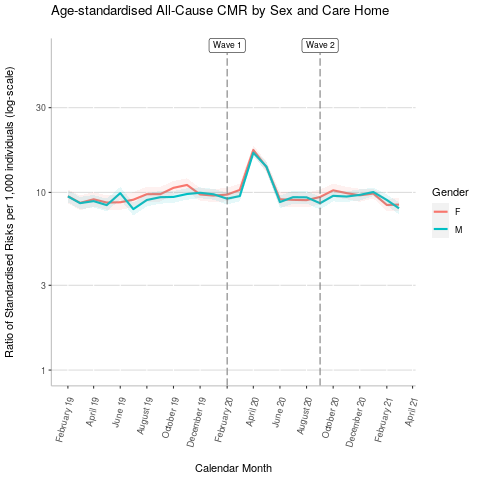


### **Figure S9a-f. Prevalence of different comorbidities over time**

1.
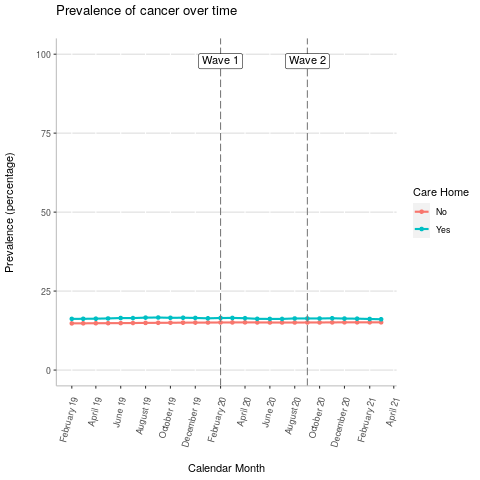

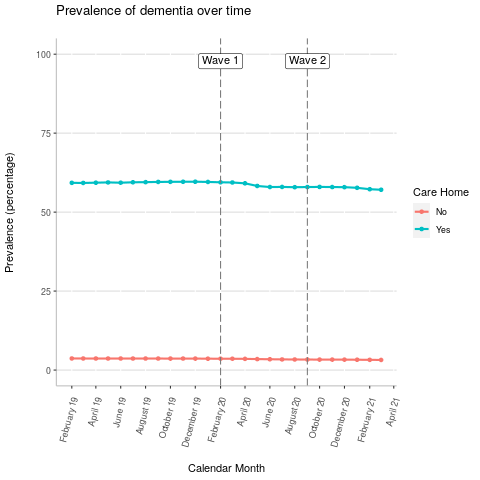
 b) c)


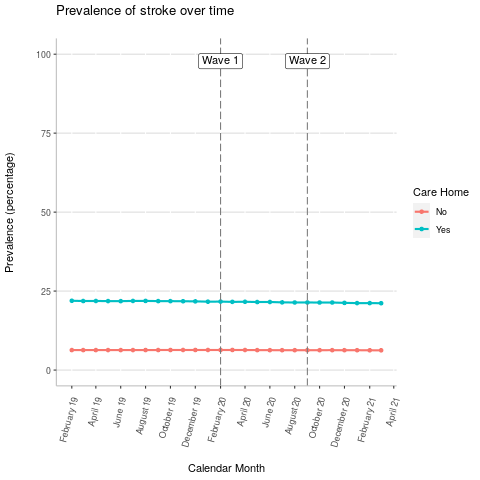


d) e) f)


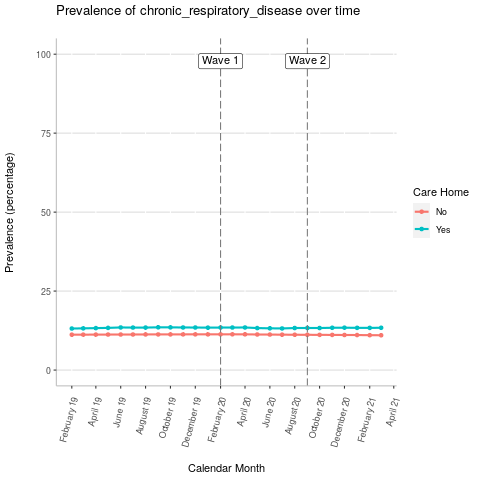

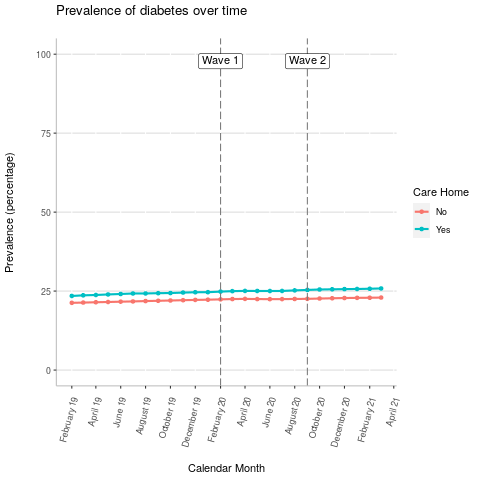

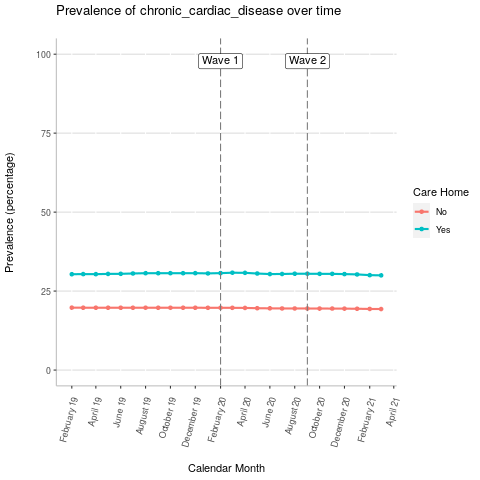


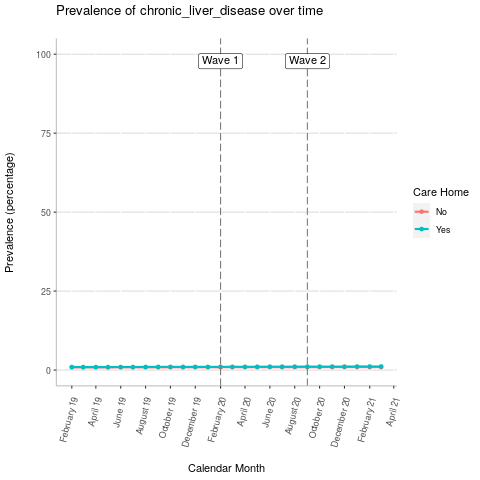
g)

### **Figure S10a-c. Age-standardised a) all-cause, b) covid and c) non-covid admission probabilities**

1. b) c)


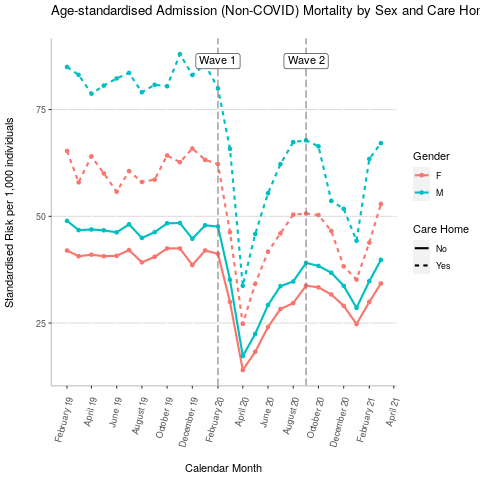

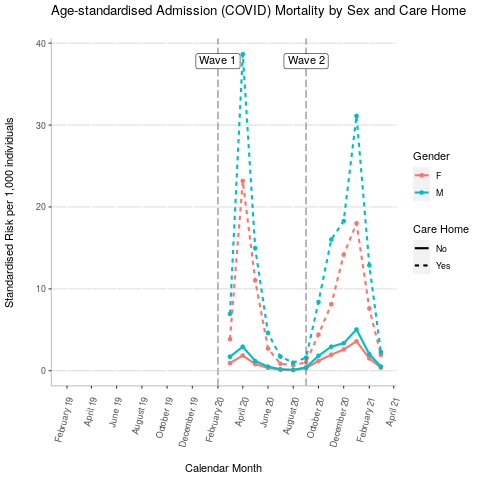

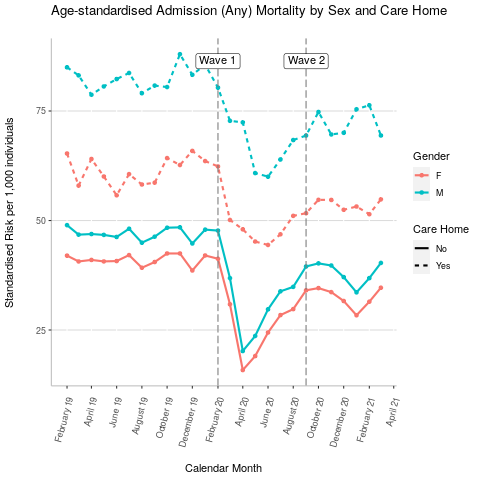


### **Figure S11a-c. Age-standardised relative a) all-cause, b) covid and c) non-covid admission probabilities**

1. b) c)


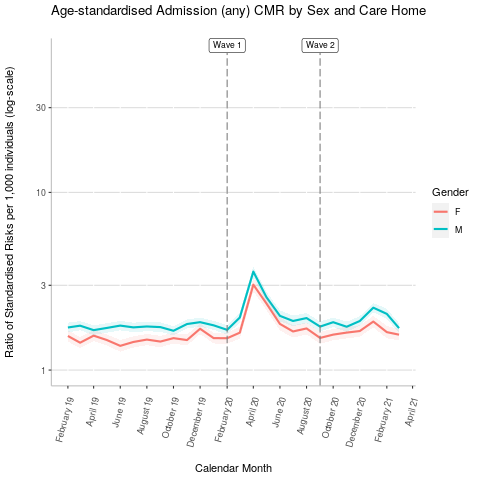

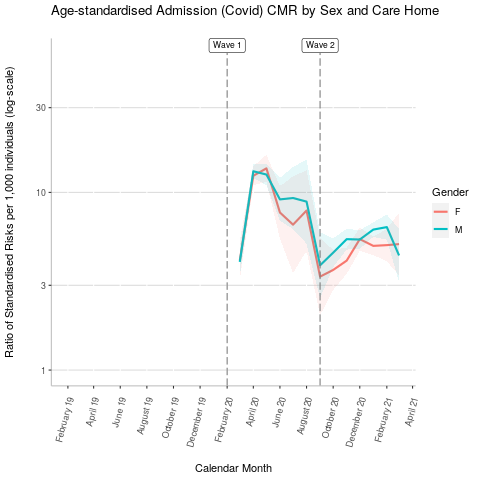

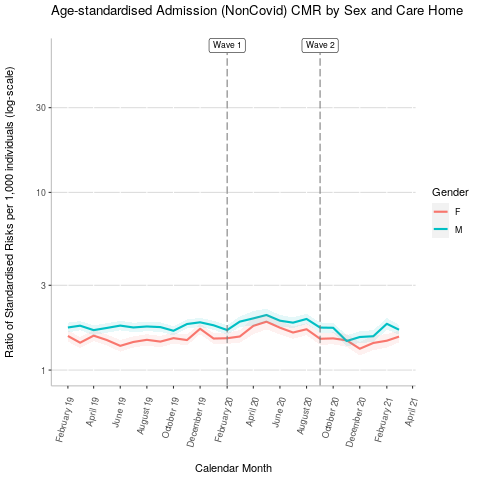


### **Figure S12a-b. Age-standardised COVID-19 a) testing probability and b) relative testing probability**

1. b)


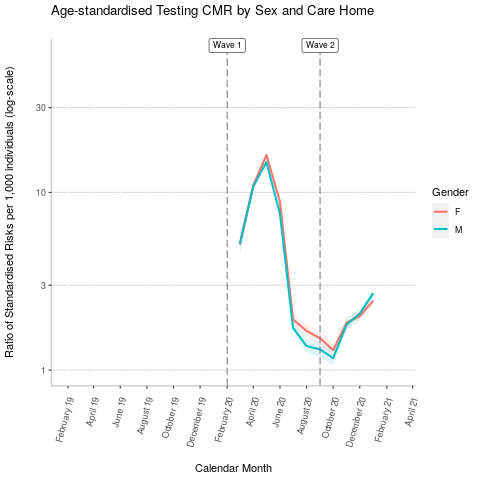


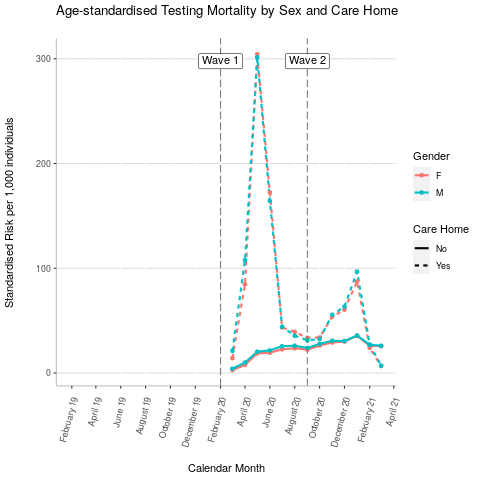


### **Figure S13. Proportion of care home deaths over time, by cause including COVID-19**

### **
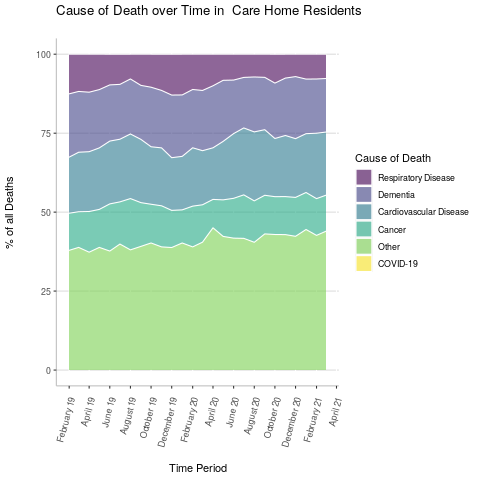
**

###

###

###

### **Table S1a. Crude all-cause mortality risks by age-group**

| **Agegroup** | **Start of Time Period** | **Care or Nursing Home** | | | | | | **Private Home** | | |
| --- | --- | --- | --- | --- | --- | --- | --- | --- | --- | --- |
| **Age** | **Date** | n | N | Mortality Risk | 95%CI | n | N | | Mortality Risk | 95% CI |
| **Total** | **Total** | **101191** | **NA** | **NA** | **NA** | **308253** | **NA** | | **NA** | **NA** |
| 65-74 | 01/02/2019 | 256 | 9578 | 28.64 | 25.37-32.31 | 2644 | 2354058 | | 1.2 | 1.16-1.25 |
| 65-74 | 01/03/2019 | 212 | 9586 | 21.4 | 18.73-24.44 | 2550 | 2354502 | | 1.05 | 1.01-1.09 |
| 65-74 | 01/04/2019 | 247 | 9611 | 25.7 | 22.72-29.06 | 2586 | 2354321 | | 1.1 | 1.06-1.14 |
| 65-74 | 01/05/2019 | 171 | 9646 | 17.16 | 14.79-19.9 | 2469 | 2355810 | | 1.01 | 0.98-1.06 |
| 65-74 | 01/06/2019 | 210 | 9757 | 21.52 | 18.83-24.6 | 2436 | 2356276 | | 1.03 | 0.99-1.08 |
| 65-74 | 01/07/2019 | 196 | 9811 | 19.33 | 16.83-22.2 | 2429 | 2356577 | | 1 | 0.96-1.04 |
| 65-74 | 01/08/2019 | 218 | 9929 | 21.25 | 18.63-24.22 | 2453 | 2358366 | | 1.01 | 0.97-1.05 |
| 65-74 | 01/09/2019 | 227 | 10005 | 22.69 | 19.95-25.79 | 2414 | 2359740 | | 1.02 | 0.98-1.06 |
| 65-74 | 01/10/2019 | 236 | 10012 | 22.81 | 20.11-25.87 | 2522 | 2359824 | | 1.03 | 0.99-1.08 |
| 65-74 | 01/11/2019 | 264 | 10114 | 26.1 | 23.17-29.39 | 2618 | 2360599 | | 1.11 | 1.07-1.15 |
| 65-74 | 01/12/2019 | 281 | 10099 | 26.93 | 23.99-30.21 | 2893 | 2361157 | | 1.19 | 1.14-1.23 |
| 65-74 | 01/01/2020 | 275 | 10142 | 26.24 | 23.35-29.48 | 2905 | 2362671 | | 1.19 | 1.15-1.23 |
| 65-74 | 01/02/2020 | 236 | 10215 | 23.9 | 21.07-27.1 | 2549 | 2363592 | | 1.12 | 1.07-1.16 |
| 65-74 | 01/03/2020 | 293 | 10349 | 27.4 | 24.47-30.66 | 3028 | 2367325 | | 1.24 | 1.19-1.28 |
| 65-74 | 01/04/2020 | 649 | 10392 | 62.45 | 57.96-67.27 | 3802 | 2369480 | | 1.6 | 1.55-1.66 |
| 65-74 | 01/05/2020 | 349 | 10136 | 33.32 | 30.05-36.93 | 2767 | 2369977 | | 1.13 | 1.09-1.17 |
| 65-74 | 01/06/2020 | 196 | 10027 | 19.55 | 17.02-22.45 | 2457 | 2371772 | | 1.04 | 1-1.08 |
| 65-74 | 01/07/2020 | 201 | 10153 | 19.16 | 16.71-21.96 | 2360 | 2373489 | | 0.96 | 0.92-1 |
| 65-74 | 01/08/2020 | 234 | 10375 | 21.83 | 19.23-24.77 | 2364 | 2376068 | | 0.96 | 0.92-1 |
| 65-74 | 01/09/2020 | 198 | 10437 | 18.97 | 16.53-21.77 | 2374 | 2377926 | | 1 | 0.96-1.04 |
| 65-74 | 01/10/2020 | 258 | 10481 | 23.82 | 21.12-26.87 | 2632 | 2379759 | | 1.07 | 1.03-1.11 |
| 65-74 | 01/11/2020 | 269 | 10542 | 25.52 | 22.68-28.7 | 2826 | 2381486 | | 1.19 | 1.14-1.23 |
| 65-74 | 01/12/2020 | 235 | 10550 | 21.56 | 18.99-24.45 | 2389 | 2382017 | | 0.97 | 0.93-1.01 |
| 65-74 | 01/01/2021 | 412 | 10551 | 37.79 | 34.37-41.53 | 4278 | 2384622 | | 1.74 | 1.68-1.79 |
| 65-74 | 01/02/2021 | 238 | 10415 | 24.48 | 21.59-27.75 | 3034 | 2385699 | | 1.36 | 1.31-1.41 |
| 65-74 | 01/03/2021 | 203 | 10440 | 18.82 | 16.42-21.56 | 2585 | 2386063 | | 1.05 | 1.01-1.09 |
| 75-79 | 01/02/2019 | 302 | 10077 | 32.11 | 28.73-35.87 | 1785 | 789762 | | 2.42 | 2.31-2.54 |
| 75-79 | 01/03/2019 | 276 | 10079 | 26.5 | 23.59-29.76 | 1750 | 794227 | | 2.13 | 2.03-2.23 |
| 75-79 | 01/04/2019 | 266 | 10143 | 26.22 | 23.29-29.52 | 1764 | 798291 | | 2.21 | 2.11-2.32 |
| 75-79 | 01/05/2019 | 269 | 10193 | 25.54 | 22.7-28.73 | 1676 | 802968 | | 2.02 | 1.93-2.12 |
| 75-79 | 01/06/2019 | 263 | 10321 | 25.48 | 22.61-28.7 | 1605 | 807408 | | 1.99 | 1.89-2.09 |
| 75-79 | 01/07/2019 | 261 | 10438 | 24.2 | 21.46-27.27 | 1703 | 811618 | | 2.03 | 1.94-2.13 |
| 75-79 | 01/08/2019 | 252 | 10549 | 23.12 | 20.46-26.11 | 1615 | 814633 | | 1.92 | 1.83-2.01 |
| 75-79 | 01/09/2019 | 277 | 10663 | 25.98 | 23.12-29.17 | 1664 | 818155 | | 2.03 | 1.94-2.13 |
| 75-79 | 01/10/2019 | 316 | 10675 | 28.65 | 25.7-31.93 | 1816 | 821989 | | 2.14 | 2.04-2.24 |
| 75-79 | 01/11/2019 | 362 | 10816 | 33.47 | 30.24-37.03 | 1910 | 825876 | | 2.31 | 2.21-2.42 |
| 75-79 | 01/12/2019 | 400 | 10805 | 35.83 | 32.54-39.43 | 2176 | 830754 | | 2.53 | 2.43-2.64 |
| 75-79 | 01/01/2020 | 395 | 10785 | 35.44 | 32.17-39.04 | 2132 | 833155 | | 2.48 | 2.37-2.58 |
| 75-79 | 01/02/2020 | 318 | 10863 | 30.28 | 27.17-33.74 | 1779 | 834517 | | 2.21 | 2.11-2.31 |
| 75-79 | 01/03/2020 | 393 | 10922 | 34.82 | 31.6-38.36 | 2169 | 836017 | | 2.51 | 2.41-2.62 |
| 75-79 | 01/04/2020 | 931 | 11008 | 84.57 | 79.52-89.92 | 2880 | 837081 | | 3.44 | 3.32-3.57 |
| 75-79 | 01/05/2020 | 519 | 10486 | 47.9 | 44.04-52.08 | 1938 | 838417 | | 2.24 | 2.14-2.34 |
| 75-79 | 01/06/2020 | 269 | 10245 | 26.26 | 23.33-29.54 | 1713 | 841298 | | 2.04 | 1.94-2.13 |
| 75-79 | 01/07/2020 | 270 | 10432 | 25.05 | 22.26-28.17 | 1651 | 843878 | | 1.89 | 1.8-1.99 |
| 75-79 | 01/08/2020 | 232 | 10635 | 21.11 | 18.59-23.97 | 1699 | 846641 | | 1.94 | 1.85-2.04 |
| 75-79 | 01/09/2020 | 258 | 10743 | 24.02 | 21.29-27.09 | 1714 | 848193 | | 2.02 | 1.93-2.12 |
| 75-79 | 01/10/2020 | 335 | 10881 | 29.79 | 26.81-33.1 | 1956 | 850699 | | 2.23 | 2.13-2.33 |
| 75-79 | 01/11/2020 | 393 | 10989 | 35.76 | 32.45-39.4 | 2025 | 854441 | | 2.37 | 2.27-2.48 |
| 75-79 | 01/12/2020 | 316 | 10960 | 27.9 | 25.03-31.1 | 1861 | 857153 | | 2.1 | 2.01-2.2 |
| 75-79 | 01/01/2021 | 553 | 10898 | 49.11 | 45.27-53.25 | 3179 | 860044 | | 3.58 | 3.46-3.7 |
| 75-79 | 01/02/2021 | 333 | 10816 | 32.99 | 29.67-36.66 | 2182 | 861894 | | 2.71 | 2.6-2.83 |
| 75-79 | 01/03/2021 | 285 | 10837 | 25.45 | 22.69-28.53 | 1844 | 865811 | | 2.06 | 1.97-2.16 |
| 80-84 | 01/02/2019 | 583 | 17541 | 35.61 | 32.88-38.56 | 2304 | 580377 | | 4.25 | 4.08-4.43 |
| 80-84 | 01/03/2019 | 515 | 17563 | 28.38 | 26.06-30.89 | 2194 | 581608 | | 3.65 | 3.5-3.81 |
| 80-84 | 01/04/2019 | 528 | 17562 | 30.06 | 27.64-32.7 | 2236 | 583123 | | 3.83 | 3.68-4 |
| 80-84 | 01/05/2019 | 546 | 17661 | 29.92 | 27.54-32.49 | 2127 | 585079 | | 3.52 | 3.37-3.67 |
| 80-84 | 01/06/2019 | 533 | 17920 | 29.74 | 27.36-32.33 | 1910 | 586571 | | 3.26 | 3.11-3.41 |
| 80-84 | 01/07/2019 | 507 | 17940 | 27.35 | 25.1-29.8 | 2099 | 588110 | | 3.45 | 3.31-3.6 |
| 80-84 | 01/08/2019 | 527 | 18128 | 28.13 | 25.86-30.6 | 1957 | 589400 | | 3.21 | 3.07-3.36 |
| 80-84 | 01/09/2019 | 537 | 18251 | 29.42 | 27.07-31.98 | 1970 | 590816 | | 3.33 | 3.19-3.48 |
| 80-84 | 01/10/2019 | 648 | 18224 | 34.41 | 31.9-37.11 | 2225 | 591329 | | 3.64 | 3.49-3.8 |
| 80-84 | 01/11/2019 | 636 | 18267 | 34.82 | 32.25-37.57 | 2332 | 591606 | | 3.94 | 3.79-4.1 |
| 80-84 | 01/12/2019 | 727 | 18180 | 38.7 | 36.04-41.55 | 2608 | 592129 | | 4.26 | 4.1-4.43 |
| 80-84 | 01/01/2020 | 683 | 18117 | 36.48 | 33.89-39.26 | 2529 | 592584 | | 4.13 | 3.97-4.29 |
| 80-84 | 01/02/2020 | 622 | 18332 | 35.1 | 32.49-37.91 | 2163 | 593149 | | 3.77 | 3.62-3.93 |
| 80-84 | 01/03/2020 | 718 | 18411 | 37.74 | 35.13-40.54 | 2578 | 594164 | | 4.2 | 4.04-4.36 |
| 80-84 | 01/04/2020 | 1717 | 18508 | 92.77 | 88.68-97.04 | 3527 | 594306 | | 5.93 | 5.74-6.13 |
| 80-84 | 01/05/2020 | 922 | 17495 | 51 | 47.89-54.3 | 2435 | 593421 | | 3.97 | 3.82-4.13 |
| 80-84 | 01/06/2020 | 497 | 17061 | 29.13 | 26.71-31.76 | 2033 | 592413 | | 3.43 | 3.29-3.58 |
| 80-84 | 01/07/2020 | 468 | 17150 | 26.41 | 24.15-28.87 | 1925 | 592254 | | 3.15 | 3.01-3.29 |
| 80-84 | 01/08/2020 | 514 | 17562 | 28.32 | 26.01-30.84 | 2061 | 592728 | | 3.36 | 3.22-3.51 |
| 80-84 | 01/09/2020 | 495 | 17629 | 28.08 | 25.74-30.62 | 2011 | 594035 | | 3.39 | 3.24-3.54 |
| 80-84 | 01/10/2020 | 616 | 17712 | 33.66 | 31.14-36.37 | 2253 | 593975 | | 3.67 | 3.52-3.83 |
| 80-84 | 01/11/2020 | 709 | 17791 | 39.85 | 37.08-42.83 | 2541 | 593239 | | 4.28 | 4.12-4.45 |
| 80-84 | 01/12/2020 | 620 | 17606 | 34.08 | 31.54-36.81 | 2198 | 592879 | | 3.59 | 3.44-3.74 |
| 80-84 | 01/01/2021 | 1106 | 17475 | 61.25 | 57.85-64.84 | 3785 | 592839 | | 6.18 | 5.99-6.38 |
| 80-84 | 01/02/2021 | 656 | 17104 | 41.09 | 38.12-44.29 | 2569 | 590077 | | 4.66 | 4.49-4.85 |
| 80-84 | 01/03/2021 | 474 | 16958 | 27.05 | 24.75-29.56 | 2133 | 588305 | | 3.51 | 3.36-3.66 |
| 85-89 | 01/02/2019 | 896 | 24275 | 39.55 | 37.08-42.17 | 2405 | 342414 | | 7.53 | 7.23-7.83 |
| 85-89 | 01/03/2019 | 810 | 24386 | 32.14 | 30.04-34.39 | 2191 | 342974 | | 6.18 | 5.93-6.45 |
| 85-89 | 01/04/2019 | 866 | 24413 | 35.47 | 33.22-37.87 | 2301 | 343722 | | 6.69 | 6.43-6.97 |
| 85-89 | 01/05/2019 | 795 | 24487 | 31.42 | 29.34-33.64 | 2236 | 344559 | | 6.28 | 6.03-6.54 |
| 85-89 | 01/06/2019 | 766 | 24759 | 30.94 | 28.85-33.17 | 2005 | 345157 | | 5.81 | 5.56-6.07 |
| 85-89 | 01/07/2019 | 774 | 24949 | 30.02 | 28.01-32.18 | 2031 | 345942 | | 5.68 | 5.44-5.93 |
| 85-89 | 01/08/2019 | 816 | 25224 | 31.31 | 29.26-33.49 | 2038 | 346668 | | 5.69 | 5.45-5.94 |
| 85-89 | 01/09/2019 | 790 | 25473 | 31.01 | 28.95-33.21 | 2013 | 347416 | | 5.79 | 5.55-6.05 |
| 85-89 | 01/10/2019 | 980 | 25564 | 37.1 | 34.89-39.44 | 2154 | 347834 | | 5.99 | 5.75-6.25 |
| 85-89 | 01/11/2019 | 1047 | 25593 | 40.91 | 38.55-43.41 | 2341 | 348728 | | 6.71 | 6.45-6.99 |
| 85-89 | 01/12/2019 | 1133 | 25563 | 42.89 | 40.52-45.4 | 2610 | 349395 | | 7.23 | 6.96-7.51 |
| 85-89 | 01/01/2020 | 1068 | 25404 | 40.68 | 38.36-43.14 | 2657 | 349365 | | 7.36 | 7.09-7.64 |
| 85-89 | 01/02/2020 | 996 | 25513 | 40.39 | 38-42.92 | 2368 | 348841 | | 7.02 | 6.75-7.31 |
| 85-89 | 01/03/2020 | 1092 | 25513 | 41.42 | 39.08-43.89 | 2685 | 349515 | | 7.43 | 7.16-7.72 |
| 85-89 | 01/04/2020 | 2425 | 25539 | 94.95 | 91.42-98.61 | 3716 | 349186 | | 10.64 | 10.31-10.99 |
| 85-89 | 01/05/2020 | 1362 | 24082 | 54.73 | 51.98-57.62 | 2487 | 348488 | | 6.91 | 6.64-7.18 |
| 85-89 | 01/06/2020 | 733 | 23444 | 31.27 | 29.11-33.57 | 2101 | 349307 | | 6.01 | 5.76-6.28 |
| 85-89 | 01/07/2020 | 654 | 23517 | 26.91 | 24.95-29.02 | 1953 | 349785 | | 5.4 | 5.17-5.65 |
| 85-89 | 01/08/2020 | 731 | 24007 | 29.47 | 27.44-31.64 | 1893 | 350613 | | 5.22 | 5-5.46 |
| 85-89 | 01/09/2020 | 740 | 24156 | 30.63 | 28.53-32.88 | 1926 | 351335 | | 5.48 | 5.24-5.73 |
| 85-89 | 01/10/2020 | 926 | 24220 | 37 | 34.73-39.41 | 2281 | 351874 | | 6.27 | 6.02-6.54 |
| 85-89 | 01/11/2020 | 1040 | 24352 | 42.71 | 40.24-45.32 | 2661 | 352252 | | 7.55 | 7.27-7.85 |
| 85-89 | 01/12/2020 | 917 | 24128 | 36.78 | 34.52-39.19 | 2422 | 351883 | | 6.66 | 6.4-6.93 |
| 85-89 | 01/01/2021 | 1591 | 23843 | 64.58 | 61.58-67.71 | 3884 | 351801 | | 10.68 | 10.36-11.02 |
| 85-89 | 01/02/2021 | 1047 | 23258 | 48.23 | 45.46-51.17 | 2667 | 350123 | | 8.16 | 7.86-8.48 |
| 85-89 | 01/03/2021 | 739 | 23020 | 31.07 | 28.94-33.35 | 2255 | 349927 | | 6.24 | 5.98-6.5 |
| 90+ | 01/02/2019 | 1511 | 33741 | 47.98 | 45.67-50.4 | 2506 | 178825 | | 15.01 | 14.44-15.61 |
| 90+ | 01/03/2019 | 1367 | 33799 | 39.14 | 37.16-41.22 | 2338 | 179036 | | 12.64 | 12.14-13.16 |
| 90+ | 01/04/2019 | 1362 | 33999 | 40.06 | 38.03-42.2 | 2338 | 179219 | | 13.05 | 12.53-13.58 |
| 90+ | 01/05/2019 | 1404 | 34324 | 39.58 | 37.61-41.66 | 2229 | 180075 | | 11.98 | 11.49-12.48 |
| 90+ | 01/06/2019 | 1269 | 34755 | 36.51 | 34.59-38.54 | 1994 | 180484 | | 11.05 | 10.58-11.54 |
| 90+ | 01/07/2019 | 1347 | 35096 | 37.14 | 35.25-39.14 | 1979 | 181084 | | 10.58 | 10.12-11.05 |
| 90+ | 01/08/2019 | 1316 | 35530 | 35.84 | 33.99-37.79 | 2026 | 181650 | | 10.79 | 10.34-11.27 |
| 90+ | 01/09/2019 | 1310 | 35915 | 36.48 | 34.59-38.46 | 2043 | 182412 | | 11.2 | 10.73-11.69 |
| 90+ | 01/10/2019 | 1605 | 36085 | 43.04 | 41.03-45.15 | 2347 | 182568 | | 12.44 | 11.95-12.95 |
| 90+ | 01/11/2019 | 1665 | 36278 | 45.9 | 43.79-48.1 | 2426 | 182385 | | 13.3 | 12.79-13.84 |
| 90+ | 01/12/2019 | 1871 | 36271 | 49.92 | 47.76-52.17 | 2837 | 182451 | | 15.05 | 14.51-15.61 |
| 90+ | 01/01/2020 | 1806 | 36159 | 48.33 | 46.21-50.55 | 2867 | 182412 | | 15.21 | 14.67-15.77 |
| 90+ | 01/02/2020 | 1610 | 36124 | 46.11 | 43.95-48.36 | 2409 | 181593 | | 13.72 | 13.19-14.28 |
| 90+ | 01/03/2020 | 1763 | 36381 | 46.9 | 44.81-49.08 | 2812 | 182318 | | 14.93 | 14.39-15.48 |
| 90+ | 01/04/2020 | 3472 | 36573 | 94.93 | 91.97-97.98 | 3736 | 181913 | | 20.54 | 19.9-21.2 |
| 90+ | 01/05/2020 | 2291 | 34710 | 63.87 | 61.39-66.45 | 2688 | 181343 | | 14.34 | 13.82-14.89 |
| 90+ | 01/06/2020 | 1261 | 33644 | 37.48 | 35.5-39.56 | 2051 | 181884 | | 11.28 | 10.8-11.77 |
| 90+ | 01/07/2020 | 1178 | 33822 | 33.71 | 31.87-35.65 | 1997 | 182522 | | 10.59 | 10.14-11.06 |
| 90+ | 01/08/2020 | 1288 | 34623 | 36 | 34.12-37.98 | 2017 | 183515 | | 10.64 | 10.18-11.11 |
| 90+ | 01/09/2020 | 1247 | 34855 | 35.78 | 33.88-37.78 | 2036 | 184289 | | 11.05 | 10.58-11.54 |
| 90+ | 01/10/2020 | 1491 | 35198 | 40.99 | 39.01-43.08 | 2455 | 184658 | | 12.87 | 12.37-13.38 |
| 90+ | 01/11/2020 | 1719 | 35435 | 48.51 | 46.32-50.8 | 2685 | 185303 | | 14.49 | 13.96-15.04 |
| 90+ | 01/12/2020 | 1601 | 35025 | 44.24 | 42.17-46.4 | 2445 | 185358 | | 12.77 | 12.27-13.28 |
| 90+ | 01/01/2021 | 2804 | 34685 | 78.23 | 75.5-81.06 | 4155 | 185347 | | 21.69 | 21.05-22.36 |
| 90+ | 01/02/2021 | 1676 | 33612 | 53.42 | 50.99-55.97 | 2885 | 183268 | | 16.87 | 16.27-17.49 |
| 90+ | 01/03/2021 | 1311 | 33164 | 38.26 | 36.28-40.34 | 2418 | 182644 | | 12.81 | 12.31-13.33 |

### **Table S1b. Crude COVID mortality risks by age-group**

| **Agegroup** | **Start of Time Period** | **Care or Nursing Home** | | | | **Private Home** | | | |
| --- | --- | --- | --- | --- | --- | --- | --- | --- | --- |
| **Age** | **Date** | n | N | Mortality Risk | 95%CI | n | N | Mortality Risk | 95% CI |
| **Total** | **Total** | **14037** | **NA** | **NA** | **NA** | **30259** | **NA** | **NA** | **NA** |
| 65-74 | 01/02/2019 |  | 9578 |  |  |  | 2354058 |  |  |
| 65-74 | 01/03/2019 |  | 9586 |  |  |  | 2354502 |  |  |
| 65-74 | 01/04/2019 |  | 9611 |  |  |  | 2354321 |  |  |
| 65-74 | 01/05/2019 |  | 9646 |  |  |  | 2355810 |  |  |
| 65-74 | 01/06/2019 |  | 9757 |  |  |  | 2356276 |  |  |
| 65-74 | 01/07/2019 |  | 9811 |  |  |  | 2356577 |  |  |
| 65-74 | 01/08/2019 |  | 9929 |  |  |  | 2358366 |  |  |
| 65-74 | 01/09/2019 |  | 10005 |  |  |  | 2359740 |  |  |
| 65-74 | 01/10/2019 |  | 10012 |  |  |  | 2359824 |  |  |
| 65-74 | 01/11/2019 |  | 10114 |  |  |  | 2360599 |  |  |
| 65-74 | 01/12/2019 |  | 10099 |  |  |  | 2361157 |  |  |
| 65-74 | 01/01/2020 |  | 10142 |  |  |  | 2362671 |  |  |
| 65-74 | 01/02/2020 |  | 10215 |  |  |  | 2363592 |  |  |
| 65-74 | 01/03/2020 | 17 | 10349 | 1.59 | 0.99-2.54 | 258 | 2367325 | 0.11 | 0.09-0.12 |
| 65-74 | 01/04/2020 | 283 | 10392 | 27.23 | 24.27-30.54 | 1261 | 2369480 | 0.53 | 0.5-0.56 |
| 65-74 | 01/05/2020 | 141 | 10136 | 13.46 | 11.43-15.85 | 396 | 2369977 | 0.16 | 0.15-0.18 |
| 65-74 | 01/06/2020 | 25 | 10027 | 2.49 | 1.69-3.68 | 135 | 2371772 | 0.06 | 0.05-0.07 |
| 65-74 | 01/07/2020 | 14 | 10153 | 1.33 | 0.8-2.24 | 58 | 2373489 | 0.02 | 0.02-0.03 |
| 65-74 | 01/08/2020 |  | 10375 |  |  | 19 | 2376068 | 0.01 | 0-0.01 |
| 65-74 | 01/09/2020 |  | 10437 |  |  | 34 | 2377926 | 0.01 | 0.01-0.02 |
| 65-74 | 01/10/2020 | 32 | 10481 | 2.95 | 2.09-4.17 | 260 | 2379759 | 0.11 | 0.09-0.12 |
| 65-74 | 01/11/2020 | 76 | 10542 | 7.21 | 5.76-9.01 | 636 | 2381486 | 0.27 | 0.25-0.29 |
| 65-74 | 01/12/2020 | 69 | 10550 | 6.33 | 5.00 – 8.00 | 569 | 2382017 | 0.23 | 0.21-0.25 |
| 65-74 | 01/01/2021 | 201 | 10551 | 18.44 | 16.08-21.13 | 1687 | 2384622 | 0.68 | 0.65-0.72 |
| 65-74 | 01/02/2021 | 76 | 10415 | 7.82 | 6.25-9.77 | 876 | 2385699 | 0.39 | 0.37-0.42 |
| 65-74 | 01/03/2021 | 19 | 10440 | 1.76 | 1.13-2.75 | 240 | 2386063 | 0.1 | 0.09-0.11 |
| 75-79 | 01/02/2019 |  | 10077 |  |  |  | 789762 |  |  |
| 75-79 | 01/03/2019 |  | 10079 |  |  |  | 794227 |  |  |
| 75-79 | 01/04/2019 |  | 10143 |  |  |  | 798291 |  |  |
| 75-79 | 01/05/2019 |  | 10193 |  |  |  | 802968 |  |  |
| 75-79 | 01/06/2019 |  | 10321 |  |  |  | 807408 |  |  |
| 75-79 | 01/07/2019 |  | 10438 |  |  |  | 811618 |  |  |
| 75-79 | 01/08/2019 |  | 10549 |  |  |  | 814633 |  |  |
| 75-79 | 01/09/2019 |  | 10663 |  |  |  | 818155 |  |  |
| 75-79 | 01/10/2019 |  | 10675 |  |  |  | 821989 |  |  |
| 75-79 | 01/11/2019 |  | 10816 |  |  |  | 825876 |  |  |
| 75-79 | 01/12/2019 |  | 10805 |  |  |  | 830754 |  |  |
| 75-79 | 01/01/2020 |  | 10785 |  |  |  | 833155 |  |  |
| 75-79 | 01/02/2020 |  | 10863 |  |  |  | 834517 |  |  |
| 75-79 | 01/03/2020 | 22 | 10922 | 1.95 | 1.29-2.95 | 189 | 836017 | 0.22 | 0.19-0.25 |
| 75-79 | 01/04/2020 | 447 | 11008 | 40.61 | 37.08-44.46 | 1012 | 837081 | 1.21 | 1.14-1.29 |
| 75-79 | 01/05/2020 | 234 | 10486 | 21.6 | 19.02-24.51 | 298 | 838417 | 0.34 | 0.31-0.39 |
| 75-79 | 01/06/2020 | 47 | 10245 | 4.59 | 3.45-6.09 | 110 | 841298 | 0.13 | 0.11-0.16 |
| 75-79 | 01/07/2020 | 17 | 10432 | 1.58 | 0.98-2.52 | 37 | 843878 | 0.04 | 0.03-0.06 |
| 75-79 | 01/08/2020 | 6 | 10635 | 0.55 | 0.25-1.19 | 21 | 846641 | 0.02 | 0.02-0.04 |
| 75-79 | 01/09/2020 |  | 10743 |  |  | 24 | 848193 | 0.03 | 0.02-0.04 |
| 75-79 | 01/10/2020 | 28 | 10881 | 2.49 | 1.72-3.6 | 225 | 850699 | 0.26 | 0.22-0.29 |
| 75-79 | 01/11/2020 | 109 | 10989 | 9.92 | 8.23-11.95 | 504 | 854441 | 0.59 | 0.54-0.64 |
| 75-79 | 01/12/2020 | 103 | 10960 | 9.09 | 7.51-11.02 | 475 | 857153 | 0.54 | 0.49-0.59 |
| 75-79 | 01/01/2021 | 305 | 10898 | 27.08 | 24.24-30.25 | 1318 | 860044 | 1.48 | 1.41-1.57 |
| 75-79 | 01/02/2021 | 107 | 10816 | 10.6 | 8.78-12.79 | 657 | 861894 | 0.82 | 0.76-0.88 |
| 75-79 | 01/03/2021 | 25 | 10837 | 2.23 | 1.51-3.29 | 168 | 865811 | 0.19 | 0.16-0.22 |
| 80-84 | 01/02/2019 |  | 17541 |  |  |  | 580377 |  |  |
| 80-84 | 01/03/2019 |  | 17563 |  |  |  | 581608 |  |  |
| 80-84 | 01/04/2019 |  | 17562 |  |  |  | 583123 |  |  |
| 80-84 | 01/05/2019 |  | 17661 |  |  |  | 585079 |  |  |
| 80-84 | 01/06/2019 |  | 17920 |  |  |  | 586571 |  |  |
| 80-84 | 01/07/2019 |  | 17940 |  |  |  | 588110 |  |  |
| 80-84 | 01/08/2019 |  | 18128 |  |  |  | 589400 |  |  |
| 80-84 | 01/09/2019 |  | 18251 |  |  |  | 590816 |  |  |
| 80-84 | 01/10/2019 |  | 18224 |  |  |  | 591329 |  |  |
| 80-84 | 01/11/2019 |  | 18267 |  |  |  | 591606 |  |  |
| 80-84 | 01/12/2019 |  | 18180 |  |  |  | 592129 |  |  |
| 80-84 | 01/01/2020 |  | 18117 |  |  |  | 592584 |  |  |
| 80-84 | 01/02/2020 |  | 18332 |  |  |  | 593149 |  |  |
| 80-84 | 01/03/2020 | 37 | 18411 | 1.94 | 1.41-2.68 | 229 | 594164 | 0.37 | 0.33-0.42 |
| 80-84 | 01/04/2020 | 769 | 18508 | 41.55 | 38.77-44.52 | 1269 | 594306 | 2.14 | 2.02-2.26 |
| 80-84 | 01/05/2020 | 410 | 17495 | 22.68 | 20.61-24.95 | 481 | 593421 | 0.78 | 0.72-0.86 |
| 80-84 | 01/06/2020 | 103 | 17061 | 6.04 | 4.98-7.32 | 162 | 592413 | 0.27 | 0.23-0.32 |
| 80-84 | 01/07/2020 | 27 | 17150 | 1.52 | 1.05-2.22 | 39 | 592254 | 0.06 | 0.05-0.09 |
| 80-84 | 01/08/2020 | 8 | 17562 | 0.44 | 0.22-0.87 | 17 | 592728 | 0.03 | 0.02-0.04 |
| 80-84 | 01/09/2020 | 8 | 17629 | 0.45 | 0.23-0.9 | 48 | 594035 | 0.08 | 0.06-0.11 |
| 80-84 | 01/10/2020 | 60 | 17712 | 3.28 | 2.55-4.22 | 255 | 593975 | 0.42 | 0.37-0.47 |
| 80-84 | 01/11/2020 | 207 | 17791 | 11.64 | 10.16-13.32 | 688 | 593239 | 1.16 | 1.08-1.25 |
| 80-84 | 01/12/2020 | 251 | 17606 | 13.8 | 12.2-15.6 | 611 | 592879 | 1 | 0.92-1.08 |
| 80-84 | 01/01/2021 | 542 | 17475 | 30.02 | 27.63-32.6 | 1620 | 592839 | 2.64 | 2.52-2.78 |
| 80-84 | 01/02/2021 | 238 | 17104 | 14.91 | 13.14-16.91 | 713 | 590077 | 1.29 | 1.2-1.39 |
| 80-84 | 01/03/2021 | 44 | 16958 | 2.51 | 1.87-3.37 | 181 | 588305 | 0.3 | 0.26-0.34 |
| 85-89 | 01/02/2019 |  | 24275 |  |  |  | 342414 |  |  |
| 85-89 | 01/03/2019 |  | 24386 |  |  |  | 342974 |  |  |
| 85-89 | 01/04/2019 |  | 24413 |  |  |  | 343722 |  |  |
| 85-89 | 01/05/2019 |  | 24487 |  |  |  | 344559 |  |  |
| 85-89 | 01/06/2019 |  | 24759 |  |  |  | 345157 |  |  |
| 85-89 | 01/07/2019 |  | 24949 |  |  |  | 345942 |  |  |
| 85-89 | 01/08/2019 |  | 25224 |  |  |  | 346668 |  |  |
| 85-89 | 01/09/2019 |  | 25473 |  |  |  | 347416 |  |  |
| 85-89 | 01/10/2019 |  | 25564 |  |  |  | 347834 |  |  |
| 85-89 | 01/11/2019 |  | 25593 |  |  |  | 348728 |  |  |
| 85-89 | 01/12/2019 |  | 25563 |  |  |  | 349395 |  |  |
| 85-89 | 01/01/2020 |  | 25404 |  |  |  | 349365 |  |  |
| 85-89 | 01/02/2020 |  | 25513 |  |  |  | 348841 |  |  |
| 85-89 | 01/03/2020 | 43 | 25513 | 1.63 | 1.21-2.2 | 195 | 349515 | 0.54 | 0.47-0.62 |
| 85-89 | 01/04/2020 | 933 | 25539 | 36.53 | 34.3-38.9 | 1269 | 349186 | 3.63 | 3.44-3.84 |
| 85-89 | 01/05/2020 | 510 | 24082 | 20.49 | 18.81-22.33 | 533 | 348488 | 1.48 | 1.36-1.61 |
| 85-89 | 01/06/2020 | 99 | 23444 | 4.22 | 3.47-5.14 | 191 | 349307 | 0.55 | 0.47-0.63 |
| 85-89 | 01/07/2020 | 30 | 23517 | 1.23 | 0.86-1.76 | 40 | 349785 | 0.11 | 0.08-0.15 |
| 85-89 | 01/08/2020 | 17 | 24007 | 0.69 | 0.43-1.1 | 15 | 350613 | 0.04 | 0.03-0.07 |
| 85-89 | 01/09/2020 | 24 | 24156 | 0.99 | 0.67-1.48 | 27 | 351335 | 0.08 | 0.05-0.11 |
| 85-89 | 01/10/2020 | 88 | 24220 | 3.52 | 2.86-4.33 | 219 | 351874 | 0.6 | 0.53-0.69 |
| 85-89 | 01/11/2020 | 287 | 24352 | 11.79 | 10.5-13.22 | 681 | 352252 | 1.93 | 1.79-2.08 |
| 85-89 | 01/12/2020 | 316 | 24128 | 12.67 | 11.36-14.14 | 632 | 351883 | 1.74 | 1.61-1.88 |
| 85-89 | 01/01/2021 | 819 | 23843 | 33.24 | 31.08-35.55 | 1635 | 351801 | 4.5 | 4.29-4.72 |
| 85-89 | 01/02/2021 | 374 | 23258 | 17.23 | 15.58-19.05 | 746 | 350123 | 2.28 | 2.12-2.45 |
| 85-89 | 01/03/2021 | 70 | 23020 | 2.94 | 2.33-3.72 | 207 | 349927 | 0.57 | 0.5-0.66 |
| 90+ | 01/02/2019 |  | 33741 |  |  |  | 178825 |  |  |
| 90+ | 01/03/2019 |  | 33799 |  |  |  | 179036 |  |  |
| 90+ | 01/04/2019 |  | 33999 |  |  |  | 179219 |  |  |
| 90+ | 01/05/2019 |  | 34324 |  |  |  | 180075 |  |  |
| 90+ | 01/06/2019 |  | 34755 |  |  |  | 180484 |  |  |
| 90+ | 01/07/2019 |  | 35096 |  |  |  | 181084 |  |  |
| 90+ | 01/08/2019 |  | 35530 |  |  |  | 181650 |  |  |
| 90+ | 01/09/2019 |  | 35915 |  |  |  | 182412 |  |  |
| 90+ | 01/10/2019 |  | 36085 |  |  |  | 182568 |  |  |
| 90+ | 01/11/2019 |  | 36278 |  |  |  | 182385 |  |  |
| 90+ | 01/12/2019 |  | 36271 |  |  |  | 182451 |  |  |
| 90+ | 01/01/2020 |  | 36159 |  |  |  | 182412 |  |  |
| 90+ | 01/02/2020 |  | 36124 |  |  |  | 181593 |  |  |
| 90+ | 01/03/2020 | 60 | 36381 | 1.6 | 1.24-2.05 | 121 | 182318 | 0.64 | 0.54-0.77 |
| 90+ | 01/04/2020 | 1181 | 36573 | 32.29 | 30.53-34.15 | 1090 | 181913 | 5.99 | 5.65-6.36 |
| 90+ | 01/05/2020 | 795 | 34710 | 22.17 | 20.69-23.74 | 554 | 181343 | 2.96 | 2.72-3.21 |
| 90+ | 01/06/2020 | 168 | 33644 | 4.99 | 4.29-5.81 | 176 | 181884 | 0.97 | 0.83-1.12 |
| 90+ | 01/07/2020 | 40 | 33822 | 1.14 | 0.84-1.56 | 36 | 182522 | 0.19 | 0.14-0.26 |
| 90+ | 01/08/2020 | 19 | 34623 | 0.53 | 0.34-0.83 | 14 | 183515 | 0.07 | 0.04-0.12 |
| 90+ | 01/09/2020 | 20 | 34855 | 0.57 | 0.37-0.89 | 25 | 184289 | 0.14 | 0.09-0.2 |
| 90+ | 01/10/2020 | 112 | 35198 | 3.08 | 2.56-3.7 | 204 | 184658 | 1.07 | 0.93-1.23 |
| 90+ | 01/11/2020 | 443 | 35435 | 12.5 | 11.4-13.71 | 579 | 185303 | 3.12 | 2.88-3.39 |
| 90+ | 01/12/2020 | 466 | 35025 | 12.88 | 11.76-14.09 | 600 | 185358 | 3.13 | 2.89-3.39 |
| 90+ | 01/01/2021 | 1346 | 34685 | 37.55 | 35.64-39.57 | 1682 | 185347 | 8.78 | 8.37-9.21 |
| 90+ | 01/02/2021 | 552 | 33612 | 17.6 | 16.2-19.11 | 789 | 183268 | 4.61 | 4.3-4.95 |
| 90+ | 01/03/2021 | 118 | 33164 | 3.44 | 2.88-4.12 | 219 | 182644 | 1.16 | 1.02-1.32 |

###

### **Table S1c. Crude non-COVID mortality rates by age-group**

| **Agegroup** | **Start of Time Period** | **Care or Nursing Home** | | | | **Private Home** | | | |
| --- | --- | --- | --- | --- | --- | --- | --- | --- | --- |
| **Age** | **Date** | n | N | Mortality Risks | 95%CI | n | N | Mortality Risks | 95% CI |
| **Total** | **Total** | **87142** | **NA** | **NA** | **NA** | **277994** | **NA** | **NA** | **NA** |
| 65-74 | 01/02/2019 | 256 | 9578 | 28.64 | 25.37-32.31 | 2644 | 2354058 | 1.2 | 1.16-1.25 |
| 65-74 | 01/03/2019 | 212 | 9586 | 21.4 | 18.73-24.44 | 2550 | 2354502 | 1.05 | 1.01-1.09 |
| 65-74 | 01/04/2019 | 247 | 9611 | 25.7 | 22.72-29.06 | 2586 | 2354321 | 1.1 | 1.06-1.14 |
| 65-74 | 01/05/2019 | 171 | 9646 | 17.16 | 14.79-19.9 | 2469 | 2355810 | 1.01 | 0.98-1.06 |
| 65-74 | 01/06/2019 | 210 | 9757 | 21.52 | 18.83-24.6 | 2436 | 2356276 | 1.03 | 0.99-1.08 |
| 65-74 | 01/07/2019 | 196 | 9811 | 19.33 | 16.83-22.2 | 2429 | 2356577 | 1 | 0.96-1.04 |
| 65-74 | 01/08/2019 | 218 | 9929 | 21.25 | 18.63-24.22 | 2453 | 2358366 | 1.01 | 0.97-1.05 |
| 65-74 | 01/09/2019 | 227 | 10005 | 22.69 | 19.95-25.79 | 2414 | 2359740 | 1.02 | 0.98-1.06 |
| 65-74 | 01/10/2019 | 236 | 10012 | 22.81 | 20.11-25.87 | 2522 | 2359824 | 1.03 | 0.99-1.08 |
| 65-74 | 01/11/2019 | 264 | 10114 | 26.1 | 23.17-29.39 | 2618 | 2360599 | 1.11 | 1.07-1.15 |
| 65-74 | 01/12/2019 | 281 | 10099 | 26.93 | 23.99-30.21 | 2893 | 2361157 | 1.19 | 1.14-1.23 |
| 65-74 | 01/01/2020 | 275 | 10142 | 26.24 | 23.35-29.48 | 2905 | 2362671 | 1.19 | 1.15-1.23 |
| 65-74 | 01/02/2020 | 236 | 10215 | 23.9 | 21.07-27.1 | 2549 | 2363592 | 1.12 | 1.07-1.16 |
| 65-74 | 01/03/2020 | 276 | 10349 | 25.81 | 22.97-28.99 | 2770 | 2367325 | 1.13 | 1.09-1.18 |
| 65-74 | 01/04/2020 | 366 | 10392 | 35.22 | 31.84-38.94 | 2541 | 2369480 | 1.07 | 1.03-1.11 |
| 65-74 | 01/05/2020 | 208 | 10136 | 19.86 | 17.36-22.71 | 2371 | 2369977 | 0.97 | 0.93-1.01 |
| 65-74 | 01/06/2020 | 171 | 10027 | 17.05 | 14.7-19.78 | 2322 | 2371772 | 0.98 | 0.94-1.02 |
| 65-74 | 01/07/2020 | 187 | 10153 | 17.82 | 15.46-20.54 | 2302 | 2373489 | 0.94 | 0.9-0.98 |
| 65-74 | 01/08/2020 | 229 | 10375 | 21.36 | 18.79-24.27 | 2345 | 2376068 | 0.96 | 0.92-0.99 |
| 65-74 | 01/09/2020 | 194 | 10437 | 18.59 | 16.17-21.36 | 2340 | 2377926 | 0.98 | 0.94-1.02 |
| 65-74 | 01/10/2020 | 226 | 10481 | 20.87 | 18.34-23.73 | 2372 | 2379759 | 0.96 | 0.93-1 |
| 65-74 | 01/11/2020 | 193 | 10542 | 18.31 | 15.92-21.05 | 2190 | 2381486 | 0.92 | 0.88-0.96 |
| 65-74 | 01/12/2020 | 166 | 10550 | 15.23 | 13.09-17.7 | 1820 | 2382017 | 0.74 | 0.71-0.77 |
| 65-74 | 01/01/2021 | 211 | 10551 | 19.35 | 16.93-22.11 | 2591 | 2384622 | 1.05 | 1.01-1.09 |
| 65-74 | 01/02/2021 | 162 | 10415 | 16.67 | 14.3-19.41 | 2158 | 2385699 | 0.97 | 0.93-1.01 |
| 65-74 | 01/03/2021 | 184 | 10440 | 17.06 | 14.78-19.68 | 2345 | 2386063 | 0.95 | 0.91-0.99 |
| 75-79 | 01/02/2019 | 302 | 10077 | 32.11 | 28.73-35.87 | 1785 | 789762 | 2.42 | 2.31-2.54 |
| 75-79 | 01/03/2019 | 276 | 10079 | 26.5 | 23.59-29.76 | 1750 | 794227 | 2.13 | 2.03-2.23 |
| 75-79 | 01/04/2019 | 266 | 10143 | 26.22 | 23.29-29.52 | 1764 | 798291 | 2.21 | 2.11-2.32 |
| 75-79 | 01/05/2019 | 269 | 10193 | 25.54 | 22.7-28.73 | 1676 | 802968 | 2.02 | 1.93-2.12 |
| 75-79 | 01/06/2019 | 263 | 10321 | 25.48 | 22.61-28.7 | 1605 | 807408 | 1.99 | 1.89-2.09 |
| 75-79 | 01/07/2019 | 261 | 10438 | 24.2 | 21.46-27.27 | 1703 | 811618 | 2.03 | 1.94-2.13 |
| 75-79 | 01/08/2019 | 252 | 10549 | 23.12 | 20.46-26.11 | 1615 | 814633 | 1.92 | 1.83-2.01 |
| 75-79 | 01/09/2019 | 277 | 10663 | 25.98 | 23.12-29.17 | 1664 | 818155 | 2.03 | 1.94-2.13 |
| 75-79 | 01/10/2019 | 316 | 10675 | 28.65 | 25.7-31.93 | 1816 | 821989 | 2.14 | 2.04-2.24 |
| 75-79 | 01/11/2019 | 362 | 10816 | 33.47 | 30.24-37.03 | 1910 | 825876 | 2.31 | 2.21-2.42 |
| 75-79 | 01/12/2019 | 400 | 10805 | 35.83 | 32.54-39.43 | 2176 | 830754 | 2.53 | 2.43-2.64 |
| 75-79 | 01/01/2020 | 395 | 10785 | 35.44 | 32.17-39.04 | 2132 | 833155 | 2.48 | 2.37-2.58 |
| 75-79 | 01/02/2020 | 318 | 10863 | 30.28 | 27.17-33.74 | 1779 | 834517 | 2.21 | 2.11-2.31 |
| 75-79 | 01/03/2020 | 371 | 10922 | 32.87 | 29.74-36.32 | 1980 | 836017 | 2.29 | 2.19-2.4 |
| 75-79 | 01/04/2020 | 484 | 11008 | 43.97 | 40.29-47.96 | 1868 | 837081 | 2.23 | 2.13-2.33 |
| 75-79 | 01/05/2020 | 285 | 10486 | 26.3 | 23.45-29.49 | 1640 | 838417 | 1.89 | 1.8-1.99 |
| 75-79 | 01/06/2020 | 222 | 10245 | 21.67 | 19.02-24.67 | 1603 | 841298 | 1.91 | 1.81-2 |
| 75-79 | 01/07/2020 | 253 | 10432 | 23.47 | 20.78-26.5 | 1614 | 843878 | 1.85 | 1.76-1.94 |
| 75-79 | 01/08/2020 | 226 | 10635 | 20.57 | 18.08-23.39 | 1678 | 846641 | 1.92 | 1.83-2.01 |
| 75-79 | 01/09/2020 | 255 | 10743 | 23.74 | 21.02-26.79 | 1690 | 848193 | 1.99 | 1.9-2.09 |
| 75-79 | 01/10/2020 | 307 | 10881 | 27.3 | 24.45-30.48 | 1731 | 850699 | 1.97 | 1.88-2.06 |
| 75-79 | 01/11/2020 | 284 | 10989 | 25.84 | 23.04-28.98 | 1521 | 854441 | 1.78 | 1.69-1.87 |
| 75-79 | 01/12/2020 | 213 | 10960 | 18.81 | 16.46-21.48 | 1386 | 857153 | 1.56 | 1.48-1.65 |
| 75-79 | 01/01/2021 | 248 | 10898 | 22.02 | 19.47-24.9 | 1861 | 860044 | 2.09 | 2-2.19 |
| 75-79 | 01/02/2021 | 226 | 10816 | 22.39 | 19.68-25.46 | 1525 | 861894 | 1.9 | 1.8-1.99 |
| 75-79 | 01/03/2021 | 260 | 10837 | 23.22 | 20.59-26.17 | 1676 | 865811 | 1.87 | 1.79-1.97 |
| 80-84 | 01/02/2019 | 583 | 17541 | 35.61 | 32.88-38.56 | 2304 | 580377 | 4.25 | 4.08-4.43 |
| 80-84 | 01/03/2019 | 515 | 17563 | 28.38 | 26.06-30.89 | 2194 | 581608 | 3.65 | 3.5-3.81 |
| 80-84 | 01/04/2019 | 528 | 17562 | 30.06 | 27.64-32.7 | 2236 | 583123 | 3.83 | 3.68-4 |
| 80-84 | 01/05/2019 | 546 | 17661 | 29.92 | 27.54-32.49 | 2127 | 585079 | 3.52 | 3.37-3.67 |
| 80-84 | 01/06/2019 | 533 | 17920 | 29.74 | 27.36-32.33 | 1910 | 586571 | 3.26 | 3.11-3.41 |
| 80-84 | 01/07/2019 | 507 | 17940 | 27.35 | 25.1-29.8 | 2099 | 588110 | 3.45 | 3.31-3.6 |
| 80-84 | 01/08/2019 | 527 | 18128 | 28.13 | 25.86-30.6 | 1957 | 589400 | 3.21 | 3.07-3.36 |
| 80-84 | 01/09/2019 | 537 | 18251 | 29.42 | 27.07-31.98 | 1970 | 590816 | 3.33 | 3.19-3.48 |
| 80-84 | 01/10/2019 | 648 | 18224 | 34.41 | 31.9-37.11 | 2225 | 591329 | 3.64 | 3.49-3.8 |
| 80-84 | 01/11/2019 | 636 | 18267 | 34.82 | 32.25-37.57 | 2332 | 591606 | 3.94 | 3.79-4.1 |
| 80-84 | 01/12/2019 | 727 | 18180 | 38.7 | 36.04-41.55 | 2608 | 592129 | 4.26 | 4.1-4.43 |
| 80-84 | 01/01/2020 | 683 | 18117 | 36.48 | 33.89-39.26 | 2529 | 592584 | 4.13 | 3.97-4.29 |
| 80-84 | 01/02/2020 | 622 | 18332 | 35.1 | 32.49-37.91 | 2163 | 593149 | 3.77 | 3.62-3.93 |
| 80-84 | 01/03/2020 | 681 | 18411 | 35.8 | 33.25-38.53 | 2349 | 594164 | 3.83 | 3.67-3.98 |
| 80-84 | 01/04/2020 | 948 | 18508 | 51.22 | 48.14-54.49 | 2258 | 594306 | 3.8 | 3.65-3.96 |
| 80-84 | 01/05/2020 | 512 | 17495 | 28.32 | 26-30.84 | 1954 | 593421 | 3.19 | 3.05-3.33 |
| 80-84 | 01/06/2020 | 394 | 17061 | 23.09 | 20.94-25.46 | 1871 | 592413 | 3.16 | 3.02-3.3 |
| 80-84 | 01/07/2020 | 441 | 17150 | 24.88 | 22.69-27.28 | 1886 | 592254 | 3.08 | 2.95-3.22 |
| 80-84 | 01/08/2020 | 506 | 17562 | 27.88 | 25.59-30.38 | 2044 | 592728 | 3.34 | 3.2-3.48 |
| 80-84 | 01/09/2020 | 487 | 17629 | 27.62 | 25.31-30.15 | 1963 | 594035 | 3.3 | 3.16-3.45 |
| 80-84 | 01/10/2020 | 556 | 17712 | 30.38 | 27.99-32.96 | 1998 | 593975 | 3.26 | 3.12-3.4 |
| 80-84 | 01/11/2020 | 502 | 17791 | 28.22 | 25.88-30.75 | 1853 | 593239 | 3.12 | 2.98-3.27 |
| 80-84 | 01/12/2020 | 369 | 17606 | 20.28 | 18.33-22.43 | 1587 | 592879 | 2.59 | 2.47-2.72 |
| 80-84 | 01/01/2021 | 564 | 17475 | 31.23 | 28.8-33.87 | 2165 | 592839 | 3.53 | 3.39-3.69 |
| 80-84 | 01/02/2021 | 418 | 17104 | 26.18 | 23.82-28.78 | 1856 | 590077 | 3.37 | 3.22-3.53 |
| 80-84 | 01/03/2021 | 430 | 16958 | 24.54 | 22.35-26.93 | 1952 | 588305 | 3.21 | 3.07-3.36 |
| 85-89 | 01/02/2019 | 896 | 24275 | 39.55 | 37.08-42.17 | 2405 | 342414 | 7.53 | 7.23-7.83 |
| 85-89 | 01/03/2019 | 810 | 24386 | 32.14 | 30.04-34.39 | 2191 | 342974 | 6.18 | 5.93-6.45 |
| 85-89 | 01/04/2019 | 866 | 24413 | 35.47 | 33.22-37.87 | 2301 | 343722 | 6.69 | 6.43-6.97 |
| 85-89 | 01/05/2019 | 795 | 24487 | 31.42 | 29.34-33.64 | 2236 | 344559 | 6.28 | 6.03-6.54 |
| 85-89 | 01/06/2019 | 766 | 24759 | 30.94 | 28.85-33.17 | 2005 | 345157 | 5.81 | 5.56-6.07 |
| 85-89 | 01/07/2019 | 774 | 24949 | 30.02 | 28.01-32.18 | 2031 | 345942 | 5.68 | 5.44-5.93 |
| 85-89 | 01/08/2019 | 816 | 25224 | 31.31 | 29.26-33.49 | 2038 | 346668 | 5.69 | 5.45-5.94 |
| 85-89 | 01/09/2019 | 790 | 25473 | 31.01 | 28.95-33.21 | 2013 | 347416 | 5.79 | 5.55-6.05 |
| 85-89 | 01/10/2019 | 980 | 25564 | 37.1 | 34.89-39.44 | 2154 | 347834 | 5.99 | 5.75-6.25 |
| 85-89 | 01/11/2019 | 1047 | 25593 | 40.91 | 38.55-43.41 | 2341 | 348728 | 6.71 | 6.45-6.99 |
| 85-89 | 01/12/2019 | 1133 | 25563 | 42.89 | 40.52-45.4 | 2610 | 349395 | 7.23 | 6.96-7.51 |
| 85-89 | 01/01/2020 | 1068 | 25404 | 40.68 | 38.36-43.14 | 2657 | 349365 | 7.36 | 7.09-7.64 |
| 85-89 | 01/02/2020 | 996 | 25513 | 40.39 | 38-42.92 | 2368 | 348841 | 7.02 | 6.75-7.31 |
| 85-89 | 01/03/2020 | 1049 | 25513 | 39.79 | 37.5-42.22 | 2490 | 349515 | 6.89 | 6.63-7.17 |
| 85-89 | 01/04/2020 | 1492 | 25539 | 58.42 | 55.61-61.36 | 2447 | 349186 | 7.01 | 6.74-7.29 |
| 85-89 | 01/05/2020 | 852 | 24082 | 34.24 | 32.05-36.57 | 1954 | 348488 | 5.43 | 5.19-5.67 |
| 85-89 | 01/06/2020 | 634 | 23444 | 27.04 | 25.04-29.2 | 1910 | 349307 | 5.47 | 5.23-5.72 |
| 85-89 | 01/07/2020 | 624 | 23517 | 25.68 | 23.76-27.74 | 1913 | 349785 | 5.29 | 5.06-5.53 |
| 85-89 | 01/08/2020 | 714 | 24007 | 28.78 | 26.77-30.94 | 1878 | 350613 | 5.18 | 4.95-5.42 |
| 85-89 | 01/09/2020 | 716 | 24156 | 29.64 | 27.58-31.86 | 1899 | 351335 | 5.41 | 5.17-5.65 |
| 85-89 | 01/10/2020 | 838 | 24220 | 33.48 | 31.33-35.78 | 2062 | 351874 | 5.67 | 5.43-5.92 |
| 85-89 | 01/11/2020 | 753 | 24352 | 30.92 | 28.82-33.17 | 1980 | 352252 | 5.62 | 5.38-5.87 |
| 85-89 | 01/12/2020 | 601 | 24128 | 24.11 | 22.27-26.08 | 1790 | 351883 | 4.92 | 4.7-5.16 |
| 85-89 | 01/01/2021 | 772 | 23843 | 31.33 | 29.23-33.58 | 2249 | 351801 | 6.19 | 5.94-6.45 |
| 85-89 | 01/02/2021 | 673 | 23258 | 31 | 28.78-33.4 | 1921 | 350123 | 5.88 | 5.62-6.15 |
| 85-89 | 01/03/2021 | 669 | 23020 | 28.12 | 26.1-30.3 | 2048 | 349927 | 5.66 | 5.42-5.91 |
| 90+ | 01/02/2019 | 1511 | 33741 | 47.98 | 45.67-50.4 | 2506 | 178825 | 15.01 | 14.44-15.61 |
| 90+ | 01/03/2019 | 1367 | 33799 | 39.14 | 37.16-41.22 | 2338 | 179036 | 12.64 | 12.14-13.16 |
| 90+ | 01/04/2019 | 1362 | 33999 | 40.06 | 38.03-42.2 | 2338 | 179219 | 13.05 | 12.53-13.58 |
| 90+ | 01/05/2019 | 1404 | 34324 | 39.58 | 37.61-41.66 | 2229 | 180075 | 11.98 | 11.49-12.48 |
| 90+ | 01/06/2019 | 1269 | 34755 | 36.51 | 34.59-38.54 | 1994 | 180484 | 11.05 | 10.58-11.54 |
| 90+ | 01/07/2019 | 1347 | 35096 | 37.14 | 35.25-39.14 | 1979 | 181084 | 10.58 | 10.12-11.05 |
| 90+ | 01/08/2019 | 1316 | 35530 | 35.84 | 33.99-37.79 | 2026 | 181650 | 10.79 | 10.34-11.27 |
| 90+ | 01/09/2019 | 1310 | 35915 | 36.48 | 34.59-38.46 | 2043 | 182412 | 11.2 | 10.73-11.69 |
| 90+ | 01/10/2019 | 1605 | 36085 | 43.04 | 41.03-45.15 | 2347 | 182568 | 12.44 | 11.95-12.95 |
| 90+ | 01/11/2019 | 1665 | 36278 | 45.9 | 43.79-48.1 | 2426 | 182385 | 13.3 | 12.79-13.84 |
| 90+ | 01/12/2019 | 1871 | 36271 | 49.92 | 47.76-52.17 | 2837 | 182451 | 15.05 | 14.51-15.61 |
| 90+ | 01/01/2020 | 1806 | 36159 | 48.33 | 46.21-50.55 | 2867 | 182412 | 15.21 | 14.67-15.77 |
| 90+ | 01/02/2020 | 1610 | 36124 | 46.11 | 43.95-48.36 | 2409 | 181593 | 13.72 | 13.19-14.28 |
| 90+ | 01/03/2020 | 1703 | 36381 | 45.3 | 43.25-47.45 | 2691 | 182318 | 14.28 | 13.76-14.83 |
| 90+ | 01/04/2020 | 2291 | 36573 | 62.64 | 60.2-65.17 | 2646 | 181913 | 14.55 | 14.01-15.11 |
| 90+ | 01/05/2020 | 1496 | 34710 | 41.71 | 39.69-43.83 | 2134 | 181343 | 11.39 | 10.92-11.88 |
| 90+ | 01/06/2020 | 1093 | 33644 | 32.49 | 30.65-34.44 | 1875 | 181884 | 10.31 | 9.85-10.78 |
| 90+ | 01/07/2020 | 1138 | 33822 | 32.56 | 30.75-34.47 | 1961 | 182522 | 10.4 | 9.95-10.87 |
| 90+ | 01/08/2020 | 1269 | 34623 | 35.47 | 33.6-37.44 | 2003 | 183515 | 10.56 | 10.11-11.03 |
| 90+ | 01/09/2020 | 1227 | 34855 | 35.2 | 33.32-37.19 | 2011 | 184289 | 10.91 | 10.45-11.4 |
| 90+ | 01/10/2020 | 1379 | 35198 | 37.91 | 36-39.93 | 2251 | 184658 | 11.8 | 11.32-12.29 |
| 90+ | 01/11/2020 | 1276 | 35435 | 36.01 | 34.12-38 | 2106 | 185303 | 11.37 | 10.89-11.86 |
| 90+ | 01/12/2020 | 1135 | 35025 | 31.36 | 29.61-33.2 | 1845 | 185358 | 9.63 | 9.21-10.08 |
| 90+ | 01/01/2021 | 1458 | 34685 | 40.68 | 38.68-42.77 | 2473 | 185347 | 12.91 | 12.42-13.43 |
| 90+ | 01/02/2021 | 1124 | 33612 | 35.83 | 33.83-37.95 | 2096 | 183268 | 12.25 | 11.74-12.79 |
| 90+ | 01/03/2021 | 1193 | 33164 | 34.81 | 32.92-36.8 | 2199 | 182644 | 11.65 | 11.18-12.15 |

###

### **Table S2a. Age-standardised all-cause mortality risks by gender**

| **Gender** | **Date** | **Care or Nursing Home Standardised Risk** | **Care or Nursing Home Confidence Interval** | **Private Home Standardised Risk** | **Private Home Confidence Interval** |
| --- | --- | --- | --- | --- | --- |
| F | 01/02/2019 | 27.79 | 24.91-30.68 | 2.62 | 2.56-2.69 |
| F | 01/03/2019 | 21.61 | 19.17-24.05 | 2.2 | 2.14-2.26 |
| F | 01/04/2019 | 23.33 | 20.91-25.74 | 2.28 | 2.22-2.35 |
| F | 01/05/2019 | 19.65 | 17.53-21.77 | 2.15 | 2.09-2.21 |
| F | 01/06/2019 | 18.91 | 16.75-21.07 | 2.03 | 1.98-2.09 |
| F | 01/07/2019 | 20.46 | 18.2-22.72 | 1.99 | 1.94-2.05 |
| F | 01/08/2019 | 21.25 | 18.82-23.68 | 2 | 1.94-2.05 |
| F | 01/09/2019 | 21.77 | 19.42-24.12 | 2.05 | 2-2.11 |
| F | 01/10/2019 | 25.22 | 22.7-27.75 | 2.17 | 2.11-2.23 |
| F | 01/11/2019 | 27.53 | 24.81-30.25 | 2.32 | 2.26-2.38 |
| F | 01/12/2019 | 27.47 | 24.9-30.03 | 2.59 | 2.53-2.65 |
| F | 01/01/2020 | 26.1 | 23.63-28.57 | 2.6 | 2.54-2.66 |
| F | 01/02/2020 | 25.02 | 22.52-27.52 | 2.35 | 2.29-2.42 |
| F | 01/03/2020 | 28.25 | 25.55-30.95 | 2.53 | 2.47-2.59 |
| F | 01/04/2020 | 59.66 | 55.97-63.36 | 3.4 | 3.32-3.47 |
| F | 01/05/2020 | 33.79 | 31.07-36.52 | 2.4 | 2.34-2.46 |
| F | 01/06/2020 | 20.58 | 18.37-22.8 | 2.11 | 2.05-2.16 |
| F | 01/07/2020 | 19.12 | 16.95-21.28 | 1.95 | 1.89-2 |
| F | 01/08/2020 | 19.22 | 17.17-21.27 | 1.96 | 1.9-2.01 |
| F | 01/09/2020 | 20.31 | 18.04-22.58 | 1.99 | 1.93-2.05 |
| F | 01/10/2020 | 24.06 | 21.67-26.46 | 2.22 | 2.16-2.28 |
| F | 01/11/2020 | 26.44 | 24.03-28.84 | 2.46 | 2.4-2.53 |
| F | 01/12/2020 | 22.06 | 19.88-24.23 | 2.16 | 2.1-2.22 |
| F | 01/01/2021 | 37.21 | 34.48-39.94 | 3.63 | 3.55-3.7 |
| F | 01/02/2021 | 24.5 | 22.3-26.69 | 2.86 | 2.79-2.93 |
| F | 01/03/2021 | 20.46 | 18.37-22.56 | 2.22 | 2.16-2.28 |
| M | 01/02/2019 | 38.69 | 35.41-41.98 | 3.56 | 3.47-3.65 |
| M | 01/03/2019 | 30.58 | 27.84-33.33 | 3.09 | 3.01-3.17 |
| M | 01/04/2019 | 31.77 | 28.99-34.54 | 3.27 | 3.18-3.36 |
| M | 01/05/2019 | 27.55 | 25.02-30.09 | 2.96 | 2.88-3.04 |
| M | 01/06/2019 | 32.88 | 29.94-35.82 | 2.83 | 2.75-2.91 |
| M | 01/07/2019 | 26.64 | 24.15-29.12 | 2.85 | 2.77-2.92 |
| M | 01/08/2019 | 28.29 | 25.77-30.8 | 2.76 | 2.68-2.84 |
| M | 01/09/2019 | 31.41 | 28.51-34.31 | 2.87 | 2.79-2.95 |
| M | 01/10/2019 | 31.51 | 28.88-34.14 | 3.05 | 2.97-3.13 |
| M | 01/11/2019 | 37.1 | 34.1-40.1 | 3.35 | 3.26-3.44 |
| M | 01/12/2019 | 40.5 | 37.39-43.62 | 3.59 | 3.5-3.68 |
| M | 01/01/2020 | 38.8 | 35.88-41.73 | 3.55 | 3.46-3.63 |
| M | 01/02/2020 | 34.33 | 31.51-37.15 | 3.31 | 3.22-3.4 |
| M | 01/03/2020 | 39.26 | 36.31-42.2 | 3.72 | 3.63-3.81 |
| M | 01/04/2020 | 94.75 | 90.25-99.25 | 5.22 | 5.11-5.32 |
| M | 01/05/2020 | 52.32 | 48.93-55.7 | 3.41 | 3.32-3.49 |
| M | 01/06/2020 | 28.73 | 26.11-31.35 | 2.89 | 2.81-2.96 |
| M | 01/07/2020 | 27.72 | 25.12-30.33 | 2.65 | 2.58-2.73 |
| M | 01/08/2020 | 29.06 | 26.42-31.7 | 2.7 | 2.62-2.77 |
| M | 01/09/2020 | 27.52 | 25.03-30.02 | 2.84 | 2.76-2.92 |
| M | 01/10/2020 | 33.81 | 31.14-36.49 | 3.18 | 3.1-3.26 |
| M | 01/11/2020 | 39.99 | 36.95-43.03 | 3.66 | 3.57-3.75 |
| M | 01/12/2020 | 33.61 | 30.92-36.3 | 3.11 | 3.03-3.19 |
| M | 01/01/2021 | 59.37 | 55.94-62.81 | 5.36 | 5.26-5.47 |
| M | 01/02/2021 | 40.35 | 37.25-43.46 | 4.03 | 3.93-4.12 |
| M | 01/03/2021 | 26.07 | 23.78-28.37 | 3.01 | 2.93-3.09 |

### **Table S2b. Age-standardised COVID mortality risks by gender**

| **Gender** | **Date** | **Care or Nursing Home Standardised Risk** | **Care or Nursing Home Confidence Interval** | **Private Home Standardised Risk** | **Private Home Confidence Interval** |
| --- | --- | --- | --- | --- | --- |
| F | 01/02/2019 |  |  |  |  |
| F | 01/03/2019 |  |  |  |  |
| F | 01/04/2019 |  |  |  |  |
| F | 01/05/2019 |  |  |  |  |
| F | 01/06/2019 |  |  |  |  |
| F | 01/07/2019 |  |  |  |  |
| F | 01/08/2019 |  |  |  |  |
| F | 01/09/2019 |  |  |  |  |
| F | 01/10/2019 |  |  |  |  |
| F | 01/11/2019 |  |  |  |  |
| F | 01/12/2019 |  |  |  |  |
| F | 01/01/2020 |  |  |  |  |
| F | 01/02/2020 |  |  |  |  |
| F | 01/03/2020 | 1.29 | 0.62-1.95 | 0.14 | 0.12-0.15 |
| F | 01/04/2020 | 24.17 | 21.7-26.63 | 0.98 | 0.94-1.02 |
| F | 01/05/2020 | 13 | 11.24-14.77 | 0.44 | 0.41-0.46 |
| F | 01/06/2020 | 3.24 | 2.27-4.2 | 0.15 | 0.13-0.16 |
| F | 01/07/2020 | 1.49 | 0.84-2.14 | 0.04 | 0.03-0.05 |
| F | 01/08/2020 | 0.37 | 0.13-0.62 | 0.02 | 0.01-0.02 |
| F | 01/09/2020 | 0.39 | 0.11-0.66 | 0.03 | 0.02-0.03 |
| F | 01/10/2020 | 2.22 | 1.48-2.96 | 0.18 | 0.17-0.2 |
| F | 01/11/2020 | 6.57 | 5.45-7.69 | 0.51 | 0.48-0.54 |
| F | 01/12/2020 | 6.36 | 5.23-7.48 | 0.49 | 0.46-0.51 |
| F | 01/01/2021 | 18.43 | 16.47-20.39 | 1.42 | 1.38-1.47 |
| F | 01/02/2021 | 7.68 | 6.49-8.86 | 0.75 | 0.71-0.78 |
| F | 01/03/2021 | 1.73 | 1.15-2.3 | 0.19 | 0.18-0.21 |
| M | 01/02/2019 |  |  |  |  |
| M | 01/03/2019 |  |  |  |  |
| M | 01/04/2019 |  |  |  |  |
| M | 01/05/2019 |  |  |  |  |
| M | 01/06/2019 |  |  |  |  |
| M | 01/07/2019 |  |  |  |  |
| M | 01/08/2019 |  |  |  |  |
| M | 01/09/2019 |  |  |  |  |
| M | 01/10/2019 |  |  |  |  |
| M | 01/11/2019 |  |  |  |  |
| M | 01/12/2019 |  |  |  |  |
| M | 01/01/2020 |  |  |  |  |
| M | 01/02/2020 |  |  |  |  |
| M | 01/03/2020 | 2.52 | 1.71-3.33 | 0.33 | 0.31-0.36 |
| M | 01/04/2020 | 45.08 | 41.89-48.26 | 1.93 | 1.86-1.99 |
| M | 01/05/2020 | 23.46 | 21.24-25.68 | 0.64 | 0.6-0.68 |
| M | 01/06/2020 | 4.68 | 3.73-5.62 | 0.23 | 0.21-0.26 |
| M | 01/07/2020 | 1.34 | 0.78-1.91 | 0.06 | 0.05-0.07 |
| M | 01/08/2020 | 0.6 | 0.21-1 | 0.02 | 0.02-0.03 |
| M | 01/09/2020 | 0.43 | 0.22-0.63 | 0.05 | 0.04-0.06 |
| M | 01/10/2020 | 3.88 | 2.88-4.88 | 0.37 | 0.34-0.4 |
| M | 01/11/2020 | 11.87 | 10.26-13.48 | 0.99 | 0.94-1.04 |
| M | 01/12/2020 | 11.69 | 10.28-13.1 | 0.88 | 0.84-0.92 |
| M | 01/01/2021 | 30.52 | 28.1-32.95 | 2.3 | 2.23-2.37 |
| M | 01/02/2021 | 13.86 | 12.07-15.64 | 1.23 | 1.17-1.28 |
| M | 01/03/2021 | 2.52 | 1.88-3.17 | 0.28 | 0.26-0.31 |

### **Table S2c. Age-standardised non-covid mortality risks by gender**

| **Gender** | **Date** | **Care or Nursing Home Standardised Risk** | **Care or Nursing Home Confidence Interval** | **Private Home Standardised Risk** | **Private Home Confidence Interval** |
| --- | --- | --- | --- | --- | --- |
| F | 01/02/2019 | 27.79 | 24.91-30.68 | 2.62 | 2.56-2.69 |
| F | 01/03/2019 | 21.61 | 19.17-24.05 | 2.2 | 2.14-2.26 |
| F | 01/04/2019 | 23.33 | 20.91-25.74 | 2.28 | 2.22-2.35 |
| F | 01/05/2019 | 19.65 | 17.53-21.77 | 2.15 | 2.09-2.21 |
| F | 01/06/2019 | 18.91 | 16.75-21.07 | 2.03 | 1.98-2.09 |
| F | 01/07/2019 | 20.46 | 18.2-22.72 | 1.99 | 1.94-2.05 |
| F | 01/08/2019 | 21.25 | 18.82-23.68 | 2 | 1.94-2.05 |
| F | 01/09/2019 | 21.77 | 19.42-24.12 | 2.05 | 2-2.11 |
| F | 01/10/2019 | 25.22 | 22.7-27.75 | 2.17 | 2.11-2.23 |
| F | 01/11/2019 | 27.53 | 24.81-30.25 | 2.32 | 2.26-2.38 |
| F | 01/12/2019 | 27.47 | 24.9-30.03 | 2.59 | 2.53-2.65 |
| F | 01/01/2020 | 26.1 | 23.63-28.57 | 2.6 | 2.54-2.66 |
| F | 01/02/2020 | 25.02 | 22.52-27.52 | 2.35 | 2.29-2.42 |
| F | 01/03/2020 | 26.96 | 24.34-29.58 | 2.4 | 2.33-2.46 |
| F | 01/04/2020 | 35.5 | 32.68-38.32 | 2.41 | 2.35-2.48 |
| F | 01/05/2020 | 20.79 | 18.69-22.89 | 1.96 | 1.91-2.02 |
| F | 01/06/2020 | 17.35 | 15.34-19.35 | 1.96 | 1.9-2.01 |
| F | 01/07/2020 | 17.63 | 15.56-19.69 | 1.91 | 1.85-1.96 |
| F | 01/08/2020 | 18.85 | 16.81-20.89 | 1.94 | 1.89-2 |
| F | 01/09/2020 | 19.93 | 17.68-22.18 | 1.96 | 1.91-2.02 |
| F | 01/10/2020 | 21.84 | 19.56-24.13 | 2.04 | 1.98-2.09 |
| F | 01/11/2020 | 19.86 | 17.72-22 | 1.95 | 1.9-2.01 |
| F | 01/12/2020 | 15.7 | 13.83-17.56 | 1.67 | 1.62-1.72 |
| F | 01/01/2021 | 18.78 | 16.85-20.71 | 2.2 | 2.15-2.26 |
| F | 01/02/2021 | 16.82 | 14.96-18.68 | 2.12 | 2.06-2.18 |
| F | 01/03/2021 | 18.74 | 16.72-20.75 | 2.03 | 1.97-2.09 |
| M | 01/02/2019 | 38.69 | 35.41-41.98 | 3.56 | 3.47-3.65 |
| M | 01/03/2019 | 30.58 | 27.84-33.33 | 3.09 | 3.01-3.17 |
| M | 01/04/2019 | 31.77 | 28.99-34.54 | 3.27 | 3.18-3.36 |
| M | 01/05/2019 | 27.55 | 25.02-30.09 | 2.96 | 2.88-3.04 |
| M | 01/06/2019 | 32.88 | 29.94-35.82 | 2.83 | 2.75-2.91 |
| M | 01/07/2019 | 26.64 | 24.15-29.12 | 2.85 | 2.77-2.92 |
| M | 01/08/2019 | 28.29 | 25.77-30.8 | 2.76 | 2.68-2.84 |
| M | 01/09/2019 | 31.41 | 28.51-34.31 | 2.87 | 2.79-2.95 |
| M | 01/10/2019 | 31.51 | 28.88-34.14 | 3.05 | 2.97-3.13 |
| M | 01/11/2019 | 37.1 | 34.1-40.1 | 3.35 | 3.26-3.44 |
| M | 01/12/2019 | 40.5 | 37.39-43.62 | 3.59 | 3.5-3.68 |
| M | 01/01/2020 | 38.8 | 35.88-41.73 | 3.55 | 3.46-3.63 |
| M | 01/02/2020 | 34.33 | 31.51-37.15 | 3.31 | 3.22-3.4 |
| M | 01/03/2020 | 36.74 | 33.9-39.58 | 3.38 | 3.3-3.47 |
| M | 01/04/2020 | 49.67 | 46.36-52.99 | 3.29 | 3.2-3.37 |
| M | 01/05/2020 | 28.86 | 26.25-31.46 | 2.77 | 2.69-2.84 |
| M | 01/06/2020 | 24.05 | 21.6-26.5 | 2.65 | 2.58-2.73 |
| M | 01/07/2020 | 26.38 | 23.84-28.92 | 2.6 | 2.52-2.67 |
| M | 01/08/2020 | 28.45 | 25.84-31.06 | 2.67 | 2.6-2.75 |
| M | 01/09/2020 | 27.1 | 24.61-29.58 | 2.79 | 2.72-2.87 |
| M | 01/10/2020 | 29.93 | 27.44-32.42 | 2.81 | 2.73-2.89 |
| M | 01/11/2020 | 28.12 | 25.52-30.72 | 2.67 | 2.6-2.75 |
| M | 01/12/2020 | 21.92 | 19.62-24.23 | 2.23 | 2.16-2.3 |
| M | 01/01/2021 | 28.85 | 26.35-31.34 | 3.06 | 2.98-3.14 |
| M | 01/02/2021 | 26.5 | 23.93-29.07 | 2.8 | 2.72-2.88 |
| M | 01/03/2021 | 23.55 | 21.35-25.75 | 2.72 | 2.65-2.8 |

### **Table S3a. Comparative Mortality Figure (all-cause) comparing Care Homes to Private Homes, by gender**

| **Gender** | **Date** | **Comparative Mortality Risk** | **Confidence Interval** |
| --- | --- | --- | --- |
| F | 01/02/2019 | 10.59 | 9.51-11.8 |
| F | 01/03/2019 | 9.82 | 8.73-11.05 |
| F | 01/04/2019 | 10.21 | 9.17-11.37 |
| F | 01/05/2019 | 9.15 | 8.18-10.24 |
| F | 01/06/2019 | 9.3 | 8.26-10.47 |
| F | 01/07/2019 | 10.26 | 9.15-11.51 |
| F | 01/08/2019 | 10.64 | 9.44-11.98 |
| F | 01/09/2019 | 10.6 | 9.47-11.86 |
| F | 01/10/2019 | 11.63 | 10.47-12.91 |
| F | 01/11/2019 | 11.88 | 10.71-13.18 |
| F | 01/12/2019 | 10.6 | 9.62-11.69 |
| F | 01/01/2020 | 10.04 | 9.09-11.08 |
| F | 01/02/2020 | 10.62 | 9.57-11.8 |
| F | 01/03/2020 | 11.16 | 10.1-12.33 |
| F | 01/04/2020 | 17.57 | 16.43-18.79 |
| F | 01/05/2020 | 14.1 | 12.94-15.36 |
| F | 01/06/2020 | 9.78 | 8.74-10.94 |
| F | 01/07/2020 | 9.81 | 8.72-11.04 |
| F | 01/08/2020 | 9.81 | 8.78-10.97 |
| F | 01/09/2020 | 10.21 | 9.09-11.47 |
| F | 01/10/2020 | 10.84 | 9.77-12.03 |
| F | 01/11/2020 | 10.73 | 9.76-11.81 |
| F | 01/12/2020 | 10.21 | 9.21-11.32 |
| F | 01/01/2021 | 10.26 | 9.49-11.08 |
| F | 01/02/2021 | 8.55 | 7.79-9.39 |
| F | 01/03/2021 | 9.2 | 8.27-10.24 |
| M | 01/02/2019 | 10.87 | 9.93-11.9 |
| M | 01/03/2019 | 9.89 | 8.99-10.87 |
| M | 01/04/2019 | 9.72 | 8.86-10.66 |
| M | 01/05/2019 | 9.3 | 8.44-10.25 |
| M | 01/06/2019 | 11.62 | 10.56-12.77 |
| M | 01/07/2019 | 9.36 | 8.48-10.32 |
| M | 01/08/2019 | 10.25 | 9.33-11.27 |
| M | 01/09/2019 | 10.94 | 9.92-12.06 |
| M | 01/10/2019 | 10.33 | 9.45-11.29 |
| M | 01/11/2019 | 11.07 | 10.16-12.07 |
| M | 01/12/2019 | 11.29 | 10.4-12.25 |
| M | 01/01/2020 | 10.94 | 10.09-11.86 |
| M | 01/02/2020 | 10.37 | 9.5-11.32 |
| M | 01/03/2020 | 10.56 | 9.75-11.44 |
| M | 01/04/2020 | 18.17 | 17.22-19.17 |
| M | 01/05/2020 | 15.35 | 14.3-16.48 |
| M | 01/06/2020 | 9.95 | 9.04-10.96 |
| M | 01/07/2020 | 10.45 | 9.46-11.54 |
| M | 01/08/2020 | 10.78 | 9.79-11.87 |
| M | 01/09/2020 | 9.68 | 8.8-10.66 |
| M | 01/10/2020 | 10.64 | 9.78-11.58 |
| M | 01/11/2020 | 10.92 | 10.07-11.84 |
| M | 01/12/2020 | 10.81 | 9.93-11.77 |
| M | 01/01/2021 | 11.08 | 10.4-11.79 |
| M | 01/02/2021 | 10.03 | 9.24-10.88 |
| M | 01/03/2021 | 8.67 | 7.9-9.51 |

### **Table S3b. Comparative Mortality Figure (covid) comparing Care Homes to Private Homes, by gender**

| **Gender** | **Date** | **Comparative Mortality Risk** | **Confidence Interval** |
| --- | --- | --- | --- |
| F | 01/02/2019 |  |  |
| F | 01/03/2019 |  |  |
| F | 01/04/2019 |  |  |
| F | 01/05/2019 |  |  |
| F | 01/06/2019 |  |  |
| F | 01/07/2019 |  |  |
| F | 01/08/2019 |  |  |
| F | 01/09/2019 |  |  |
| F | 01/10/2019 |  |  |
| F | 01/11/2019 |  |  |
| F | 01/12/2019 |  |  |
| F | 01/01/2020 |  |  |
| F | 01/02/2020 |  |  |
| F | 01/03/2020 | 9.47 | 5.59-16.04 |
| F | 01/04/2020 | 24.59 | 22.01-27.47 |
| F | 01/05/2020 | 29.83 | 25.7-34.62 |
| F | 01/06/2020 | 21.69 | 15.83-29.73 |
| F | 01/07/2020 | 37.8 | 23.38-61.11 |
| F | 01/08/2020 | 23 | 11.06-47.82 |
| F | 01/09/2020 | 14.15 | 6.71-29.81 |
| F | 01/10/2020 | 12.15 | 8.59-17.19 |
| F | 01/11/2020 | 12.83 | 10.72-15.36 |
| F | 01/12/2020 | 13.04 | 10.83-15.71 |
| F | 01/01/2021 | 12.95 | 11.57-14.48 |
| F | 01/02/2021 | 10.26 | 8.73-12.06 |
| F | 01/03/2021 | 8.96 | 6.34-12.67 |
| M | 01/02/2019 |  |  |
| M | 01/03/2019 |  |  |
| M | 01/04/2019 |  |  |
| M | 01/05/2019 |  |  |
| M | 01/06/2019 |  |  |
| M | 01/07/2019 |  |  |
| M | 01/08/2019 |  |  |
| M | 01/09/2019 |  |  |
| M | 01/10/2019 |  |  |
| M | 01/11/2019 |  |  |
| M | 01/12/2019 |  |  |
| M | 01/01/2020 |  |  |
| M | 01/02/2020 |  |  |
| M | 01/03/2020 | 7.52 | 5.4-10.49 |
| M | 01/04/2020 | 23.41 | 21.62-25.34 |
| M | 01/05/2020 | 36.63 | 32.74-40.99 |
| M | 01/06/2020 | 20.12 | 16.06-25.21 |
| M | 01/07/2020 | 23.3 | 14.68-37 |
| M | 01/08/2020 | 25.93 | 12.72-52.84 |
| M | 01/09/2020 | 8.84 | 5.25-14.87 |
| M | 01/10/2020 | 10.55 | 8.06-13.81 |
| M | 01/11/2020 | 11.98 | 10.37-13.84 |
| M | 01/12/2020 | 13.31 | 11.67-15.17 |
| M | 01/01/2021 | 13.25 | 12.16-14.45 |
| M | 01/02/2021 | 11.3 | 9.86-12.96 |
| M | 01/03/2021 | 8.92 | 6.81-11.69 |

### **Table S3c. Comparative Mortality Figure (non-covid) comparing Care Homes to Private Homes, by gender**

| **Gender** | **Date** | **Comparative Mortality Risk** | **Confidence Interval** |
| --- | --- | --- | --- |
| F | 01/02/2019 | 10.59 | 9.51-11.8 |
| F | 01/03/2019 | 9.82 | 8.73-11.05 |
| F | 01/04/2019 | 10.21 | 9.17-11.37 |
| F | 01/05/2019 | 9.15 | 8.18-10.24 |
| F | 01/06/2019 | 9.3 | 8.26-10.47 |
| F | 01/07/2019 | 10.26 | 9.15-11.51 |
| F | 01/08/2019 | 10.64 | 9.44-11.98 |
| F | 01/09/2019 | 10.6 | 9.47-11.86 |
| F | 01/10/2019 | 11.63 | 10.47-12.91 |
| F | 01/11/2019 | 11.88 | 10.71-13.18 |
| F | 01/12/2019 | 10.6 | 9.62-11.69 |
| F | 01/01/2020 | 10.04 | 9.09-11.08 |
| F | 01/02/2020 | 10.62 | 9.57-11.8 |
| F | 01/03/2020 | 11.25 | 10.17-12.46 |
| F | 01/04/2020 | 14.71 | 13.52-16.01 |
| F | 01/05/2020 | 10.6 | 9.54-11.78 |
| F | 01/06/2020 | 8.87 | 7.87-10 |
| F | 01/07/2020 | 9.23 | 8.18-10.43 |
| F | 01/08/2020 | 9.7 | 8.67-10.86 |
| F | 01/09/2020 | 10.16 | 9.03-11.42 |
| F | 01/10/2020 | 10.72 | 9.61-11.96 |
| F | 01/11/2020 | 10.18 | 9.1-11.39 |
| F | 01/12/2020 | 9.39 | 8.3-10.62 |
| F | 01/01/2021 | 8.52 | 7.66-9.48 |
| F | 01/02/2021 | 7.95 | 7.09-8.92 |
| F | 01/03/2021 | 9.23 | 8.25-10.32 |
| M | 01/02/2019 | 10.87 | 9.93-11.9 |
| M | 01/03/2019 | 9.89 | 8.99-10.87 |
| M | 01/04/2019 | 9.72 | 8.86-10.66 |
| M | 01/05/2019 | 9.3 | 8.44-10.25 |
| M | 01/06/2019 | 11.62 | 10.56-12.77 |
| M | 01/07/2019 | 9.36 | 8.48-10.32 |
| M | 01/08/2019 | 10.25 | 9.33-11.27 |
| M | 01/09/2019 | 10.94 | 9.92-12.06 |
| M | 01/10/2019 | 10.33 | 9.45-11.29 |
| M | 01/11/2019 | 11.07 | 10.16-12.07 |
| M | 01/12/2019 | 11.29 | 10.4-12.25 |
| M | 01/01/2020 | 10.94 | 10.09-11.86 |
| M | 01/02/2020 | 10.37 | 9.5-11.32 |
| M | 01/03/2020 | 10.86 | 10-11.79 |
| M | 01/04/2020 | 15.1 | 14.04-16.24 |
| M | 01/05/2020 | 10.43 | 9.48-11.47 |
| M | 01/06/2020 | 9.06 | 8.14-10.09 |
| M | 01/07/2020 | 10.16 | 9.18-11.25 |
| M | 01/08/2020 | 10.65 | 9.66-11.73 |
| M | 01/09/2020 | 9.7 | 8.8-10.68 |
| M | 01/10/2020 | 10.66 | 9.75-11.64 |
| M | 01/11/2020 | 10.52 | 9.54-11.6 |
| M | 01/12/2020 | 9.83 | 8.8-10.98 |
| M | 01/01/2021 | 9.43 | 8.61-10.34 |
| M | 01/02/2021 | 9.47 | 8.55-10.48 |
| M | 01/03/2021 | 8.65 | 7.83-9.54 |

| **Table S4. Demographic and Clinical Characteristics of Care Home Residents over time** | | | | | | | | | |  |  |
| --- | --- | --- | --- | --- | --- | --- | --- | --- | --- | --- | --- |
|  |  | **Feb-19** | | **Feb-20** | | **Sep-20** | | **Feb-21** | | | |
|  |  | **N** | **%** | **N** | **%** | **N** | **%** | **N** | **%** | |  |
| **Total** |  | 95212 | 100 | 101047 | 100 | 97820 | 100 | 95205 | 100 | |  |
| Care Home Type | Care Home | 49227 | 51.7 | 52662 | 52.12 | 51605 | 52.76 | 50115 | 52.64 | |  |
|  | Care or Nursing Home | 2352 | 2.47 | 2413 | 2.39 | 2352 | 2.4 | 2115 | 2.22 | |  |
|  | Nursing Home | 43633 | 45.83 | 45972 | 45.5 | 43863 | 44.84 | 42975 | 45.14 | |  |
|  | Private Home |  |  |  |  |  |  |  |  | |  |
| Gender | Female | 66574 | 69.92 | 70622 | 69.89 | 69028 | 70.57 | 67515 | 70.92 | |  |
|  | Male | 28638 | 30.08 | 30425 | 30.11 | 28792 | 29.43 | 27690 | 29.08 | |  |
| Age in Years | Mean, SD | 86 | 8 | 86 | 8 | 86 | 8 | 86 | 8 | |  |
| Self - reported Ethnicity | Asian or British Asian | 556 | 0.58 | 598 | 0.59 | 607 | 0.62 | 629 | 0.66 | |  |
|  | Black | 437 | 0.46 | 485 | 0.48 | 459 | 0.47 | 449 | 0.47 | |  |
|  | Missing | 25080 | 26.34 | 24497 | 24.24 | 22585 | 23.09 | 21511 | 22.59 | |  |
|  | Mixed | 188 | 0.2 | 203 | 0.2 | 203 | 0.21 | 203 | 0.21 | |  |
|  | Other | 322 | 0.34 | 352 | 0.35 | 340 | 0.35 | 324 | 0.34 | |  |
|  | White | 68629 | 72.08 | 74912 | 74.14 | 73626 | 75.27 | 72089 | 75.72 | |  |
| Geographical Region | East | 22328 | 23.45 | 23408 | 23.17 | 22720 | 23.23 | 21866 | 22.97 | |  |
|  | East Midlands | 17112 | 17.97 | 18531 | 18.34 | 18008 | 18.41 | 17425 | 18.3 | |  |
|  | London | 1914 | 2.01 | 1847 | 1.83 | 1732 | 1.77 | 1740 | 1.83 | |  |
|  | North East | 4087 | 4.29 | 4476 | 4.43 | 4223 | 4.32 | 4172 | 4.38 | |  |
|  | North West | 9291 | 9.76 | 9912 | 9.81 | 9852 | 10.07 | 9556 | 10.04 | |  |
|  | South East | 8152 | 8.56 | 8336 | 8.25 | 7908 | 8.08 | 7677 | 8.06 | |  |
|  | South West | 15699 | 16.49 | 16990 | 16.81 | 16678 | 17.05 | 16447 | 17.28 | |  |
|  | West Midlands | 3028 | 3.18 | 3110 | 3.08 | 2978 | 3.04 | 2930 | 3.08 | |  |
|  | Yorkshire and The Humber | 13589 | 14.27 | 14425 | 14.28 | 13698 | 14 | 13367 | 14.04 | |  |
|  | Missing | 12 | 0.01 | 12 | 0.01 | 23 | 0.02 | 25 | 0.03 | |  |
| Quintile of Index of Multiple Deprivation | 1 - Least Deprived | 17284 | 18.15 | 18391 | 18.2 | 17765 | 18.16 | 17292 | 18.16 | |  |
|  | 2 | 19803 | 20.8 | 20656 | 20.44 | 20152 | 20.6 | 19622 | 20.61 | |  |
|  | 3 | 20773 | 21.82 | 22295 | 22.06 | 21542 | 22.02 | 21001 | 22.06 | |  |
|  | 4 | 19147 | 20.11 | 20463 | 20.25 | 19932 | 20.38 | 19636 | 20.62 | |  |
|  | 5 - Most Deprived | 17753 | 18.65 | 18627 | 18.43 | 17764 | 18.16 | 16986 | 17.84 | |  |
|  | Missing | 452 | 0.47 | 615 | 0.61 | 665 | 0.68 | 668 | 0.7 | |  |
| History of Stroke |  | 20884 | 21.93 | 21897 | 21.67 | 20932 | 21.4 | 20182 | 21.2 | |  |
| Dementia |  | 56433 | 59.27 | 60067 | 59.44 | 56676 | 57.94 | 54509 | 57.25 | |  |
| Diabetes |  | 22331 | 23.45 | 25094 | 24.83 | 24812 | 25.36 | 24505 | 25.74 | |  |
| Chronic Kidney Disease |  | 19579 | 20.56 | 20957 | 20.74 | 18383 | 18.79 | 17375 | 18.25 | |  |
| Cancer |  | 15424 | 16.2 | 16654 | 16.48 | 15980 | 16.34 | 15396 | 16.17 | |  |
| Chronic Liver Disease |  | 919 | 0.97 | 1021 | 1.01 | 1068 | 1.09 | 1071 | 1.12 | |  |
| Chronic Cardiac Disease |  | 28884 | 30.34 | 31009 | 30.69 | 29827 | 30.49 | 28608 | 30.05 | |  |
| Chronic Respiratory Disease |  | 12514 | 13.14 | 13597 | 13.46 | 13036 | 13.33 | 12709 | 13.35 | |  |
| New resident (not resident in prior month) |  | 4880 | 5.13 | 5744 | 5.68 | 4407 | 4.51 | 4735 | 4.97 | |  |

| **Table S5. Demographic and Clinical Characteristics of New Care Home Residents at start of 1st and 2nd Wave** | | | | | |
| --- | --- | --- | --- | --- | --- |
|  |  | **1 wave (1 Feb 2020)** | | **2 wave (1 Sep 2020)** | |
|  |  | **N** | **%** | **N** | **%** |
| **Total** |  | 5744 | 100 | 4407 | 100 |
| Care Home Type | Care Home | 2775 | 48.31 | 2012 | 45.65 |
|  | Care or Nursing Home | 128 | 2.23 | 104 | 2.36 |
|  | Nursing Home | 2841 | 49.46 | 2291 | 51.99 |
|  | Private Home |  |  |  |  |
| Gender | Female | 3592 | 62.53 | 2756 | 62.54 |
|  | Male | 2152 | 37.47 | 1651 | 37.46 |
| Age in Years | Mean, SD | 85 | 8 | 85 | 8 |
| Self - reported Ethnicity | Asian or British Asian | 41 | 0.71 | 33 | 0.75 |
|  | Black | 30 | 0.52 | 19 | 0.43 |
|  | Missing | 1291 | 22.48 | 924 | 20.97 |
|  | Mixed | 10 | 0.17 | 6 | 0.14 |
|  | Other | 13 | 0.23 | 14 | 0.32 |
|  | White | 4359 | 75.89 | 3411 | 77.4 |
| Geographical Region | East | 1348 | 23.47 | 1019 | 23.12 |
|  | East Midlands | 1164 | 20.26 | 780 | 17.7 |
|  | London | 88 | 1.53 | 111 | 2.52 |
|  | North East | 285 | 4.96 | 205 | 4.65 |
|  | North West | 614 | 10.69 | 492 | 11.16 |
|  | South East | 394 | 6.86 | 349 | 7.92 |
|  | South West | 902 | 15.7 | 669 | 15.18 |
|  | West Midlands | 150 | 2.61 | 157 | 3.56 |
|  | Yorkshire and The Humber | 799 | 13.91 | 625 | 14.18 |
|  | Missing |  |  |  |  |
| Quintile of Index of Multiple Deprivation | 1 - Least Deprived | 1093 | 19.03 | 786 | 17.84 |
|  | 2 | 1126 | 19.6 | 963 | 21.85 |
|  | 3 | 1288 | 22.42 | 926 | 21.01 |
|  | 4 | 1101 | 19.17 | 903 | 20.49 |
|  | 5 - Most Deprived | 1080 | 18.8 | 796 | 18.06 |
|  | Missing | 56 | 0.97 | 33 | 0.75 |
| History of Stroke |  | 1182 | 20.58 | 910 | 20.65 |
| Dementia |  | 2514 | 43.77 | 1930 | 43.79 |
| Diabetes |  | 1575 | 27.42 | 1298 | 29.45 |
| Chronic Kidney Disease |  | 1425 | 24.81 | 1047 | 23.76 |
| Cancer |  | 1332 | 23.19 | 1006 | 22.83 |
| Chronic Liver Disease |  | 74 | 1.29 | 70 | 1.59 |
| Chronic Cardiac Disease |  | 2027 | 35.29 | 1538 | 34.9 |
| Chronic Respiratory Disease |  | 908 | 15.81 | 694 | 15.75 |
| New resident (not resident in prior month) |  | 5744 | 100 | 4407 | 100 |
