# Supplementary material for: Mortality among Care Home Residents in England during the first and second waves of the COVID-19 pandemic: an observational study of 4.3 million adults over the age of 65
Source: Lancet Reg Health Eur. 2022 Jan 10;14:100295. doi: 10.1016/j.lanepe.2021.100295 (PMC8743167; doi:10.1016/j.lanepe.2021.100295)
Supplement: Supplementary file 2 [file mmc2.docx]

STROBE Statement—checklist of items that should be included in reports of observational studies

|  | Item No. | Recommendation | Section | Relevant text from manuscript |
| --- | --- | --- | --- | --- |
| **Title and abstract** | 1 | (*a*) Indicate the study’s design with a commonly used term in the title or the abstract | Title Page | Mortality among Care Home Residents in England during the first and second waves of the COVID-19 pandemic: an observational study of 4.3 million adults over the age of 65 |
|  |  | (*b*) Provide in the abstract an informative and balanced summary of what was done and what was found | Abstract | NA |
| Introduction | | | |  |
| Background/rationale | 2 | Explain the scientific background and rationale for the investigation being reported | Introduction | The impact of the COVID-19 pandemic on the risk of death among care home residents in England has not yet been comprehensively investigated and placed in the context of the mortality of people living in private residences, in part because of the absence of a national registry of care home residents. |
| Objectives | 3 | State specific objectives, including any prespecified hypotheses | Introduction | our aim was to provide the first direct estimates of mortality risks of care home residents compared to that of individuals in private residences across a period starting in February 2019 through waves 1 and 2 ending in March 2021 |
| Methods | | | |  |
| Study design | 4 | Present key elements of study design early in the paper | Methods | NA |
| Setting | 5 | Describe the setting, locations, and relevant dates, including periods of recruitment, exposure, follow-up, and data collection | Methods: Study Design and Population |  |
| Participants | 6 | (*a*) *Cohort study*—Give the eligibility criteria, and the sources and methods of selection of participants. Describe methods of follow-up  *Case-control study*—Give the eligibility criteria, and the sources and methods of case ascertainment and control selection. Give the rationale for the choice of cases and controls  *Cross-sectional study*—Give the eligibility criteria, and the sources and methods of selection of participants | Methods: Study Design and Population |  |
|  |  | (*b*) *Cohort study*—For matched studies, give matching criteria and number of exposed and unexposed  *Case-control study*—For matched studies, give matching criteria and the number of controls per case | NA |  |
| Variables | 7 | Clearly define all outcomes, exposures, predictors, potential confounders, and effect modifiers. Give diagnostic criteria, if applicable | Methods: Study Measures |  |
| Data sources/ measurement | 8* | For each variable of interest, give sources of data and details of methods of assessment (measurement). Describe comparability of assessment methods if there is more than one group | Methods: Study Measures |  |
| Bias | 9 | Describe any efforts to address potential sources of bias | Methods |  |
| Study size | 10 | Explain how the study size was arrived at | NA – the study did not recruit participants, all eligible individuals were included |  |

Continued on next page

| Quantitative variables | 11 | Explain how quantitative variables were handled in the analyses. If applicable, describe which groupings were chosen and why | NA – there were regression models handling quantitative variables |  |
| --- | --- | --- | --- | --- |
| Statistical methods | 12 | (*a*) Describe all statistical methods, including those used to control for confounding | Methods: Statistical Methods | To account for differences in the age of care home and private home residents, mortality risks for men and women were directly standardised to the European Standard (2013) population using five-year age-bands. |
|  |  | (*b*) Describe any methods used to examine subgroups and interactions | Methods: supplementary analyses | Finally we undertook analyses of directly standardised risks of mortality stratified into two broad age groups (up to age 80, and 80+ years) and according to whether the care home of residence was or was not a nursing home. |
|  |  | (*c*) Explain how missing data were addressed | Methods: Study Design and Population | Valid address data is missing for a small proportion of individuals aged 65 years or older registered with TPP practices (1.1%). |
|  |  | (*d*) *Cohort study*—If applicable, explain how loss to follow-up was addressed  *Case-control study*—If applicable, explain how matching of cases and controls was addressed  *Cross-sectional study*—If applicable, describe analytical methods taking account of sampling strategy | NA |  |
|  |  | (*e*) Describe any sensitivity analyses | Methods: Supplementary Analyses |  |
| Results | | | | |
| Participants | 13* | (a) Report numbers of individuals at each stage of study—eg numbers potentially eligible, examined for eligibility, confirmed eligible, included in the study, completing follow-up, and analysed | NA – exclusions were negligible and described | Valid address data is missing for a small proportion of individuals aged 65 years or older registered with TPP practices (1.1%). |
|  |  | (b) Give reasons for non-participation at each stage | NA – exclusions were negligible and described | Valid address data is missing for a small proportion of individuals aged 65 years or older registered with TPP practices (1.1%). |
|  |  | (c) Consider use of a flow diagram | NA – the study design was repeated cohorts (16 different populations) |  |
| Descriptive data | 14* | (a) Give characteristics of study participants (eg demographic, clinical, social) and information on exposures and potential confounders | Table 1 |  |
|  |  | (b) Indicate number of participants with missing data for each variable of interest | Table 1 |  |
|  |  | (c) *Cohort study*—Summarise follow-up time (eg, average and total amount) | Statistical Methods | Monthly mortality risks were calculated by totalling the number of deaths occurring during a given calendar month among people meeting the inclusion and exclusion criteria at the 1st of that specific month (numerator) and dividing these by the number of individuals meeting the inclusion and exclusion criteria at the beginning of the interval (denominator). |
| Outcome data | 15* | *Cohort study*—Report numbers of outcome events or summary measures over time | *Supplementary Materials* |  |
|  |  | *Case-control study—*Report numbers in each exposure category, or summary measures of exposure |  |  |
|  |  | Cross-sectional study—Report numbers of outcome events or summary measures |  |  |
| Main results | 16 | (a) Give unadjusted estimates and, if applicable, confounder-adjusted estimates and their precision (eg, 95% confidence interval). Make clear which confounders were adjusted for and why they were included | Results: Mortality Trends |  |
|  |  | (b) Report category boundaries when continuous variables were categorized | NA – no continuous variables in primary analyses |  |
|  |  | (c) If relevant, consider translating estimates of relative risk into absolute risk for a meaningful time period | NA – the primary outcome of interest was the relative risk |  |

Continued on next page

| Other analyses | 17 | Report other analyses done—eg analyses of subgroups and interactions, and sensitivity analyses | Results: Supplementary Analyses |  |
| --- | --- | --- | --- | --- |
| Discussion | | | | |
| Key results | 18 | Summarise key results with reference to study objectives | Discussion: summary |  |
| Limitations | 19 | Discuss limitations of the study, taking into account sources of potential bias or imprecision. Discuss both direction and magnitude of any potential bias | Discussion: Strengths and Limitations |  |
| Interpretation | 20 | Give a cautious overall interpretation of results considering objectives, limitations, multiplicity of analyses, results from similar studies, and other relevant evidence | Discussion: Interpretation and Policy Implication |  |
| Generalisability | 21 | Discuss the generalisability (external validity) of the study results |  |  |
| Other information | |  | | |
| Funding | 22 | Give the source of funding and the role of the funders for the present study and, if applicable, for the original study on which the present article is based | Methods: Role of the Funding Source, Administrative: Funding |  |

*Give information separately for cases and controls in case-control studies and, if applicable, for exposed and unexposed groups in cohort and cross-sectional studies.

**Note:** An Explanation and Elaboration article discusses each checklist item and gives methodological background and published examples of transparent reporting. The STROBE checklist is best used in conjunction with this article (freely available on the Web sites of PLoS Medicine at http://www.plosmedicine.org/, Annals of Internal Medicine at http://www.annals.org/, and Epidemiology at http://www.epidem.com/). Information on the STROBE Initiative is available at www.strobe-statement.org.
